# Methods for assessing climate vulnerability in Africa across two decades: a scoping review

**DOI:** 10.1186/s44329-025-00041-7

**Published:** 2025-12-03

**Authors:** Emily Odipo, Sharon A. Onyango, Moses C. Kiti, Robert W. Snow, Benjamin Tsofa, Jacob Mcknight, Peter M. Macharia, Emelda A. Okiro

**Affiliations:** 1 Population and Health Impact Surveillance Group, Kenya Medical Research Institute-Wellcome Trust Research Programme, Nairobi, Kenya; 2 Centre for Tropical Medicine and Global Health, Nuffield Department of Medicine, University of Oxford, Oxford, UK; 3 Health Systems and Research Ethics Department, Kenya Medical Research Institute-Wellcome Trust Research Programme, Kilifi, Kenya; 4 Department of Public Health, Institute of Tropical Medicine Antwerp, Antwerp, Belgium

**Keywords:** Climate change, Climate, Extreme weather event, Vulnerability, Africa

## Abstract

**Background:**

Climate change and extreme weather events (EWEs) have an adverse impact on both populations and their surrounding environment. These effects span regions and sectors, with varying impacts, some of which are irreversible. The changing climate, accompanied by an increasing frequency of EWEs, necessitates assessment of climate vulnerability as an important applied instrument to identify populations and systems at risk and guide decision-makers in prioritising targeted interventions. Africa exhibits considerable climatic variability and is particularly susceptible to the impacts of climate change. This review aims to identify key concepts and metrics previously used to define climate vulnerability in Africa facilitating a regional understanding of approaches across various sectors that can be adopted to understand the gaps and limitations as a basis to improve future methods.

**Methods:**

We searched literature from 1st January 2003 to 31st December 2023, restricted to publications in English. We analysed the extracted data using both descriptive and thematic approaches, consistent with established scoping review frameworks (Arksey & O’Malley, 2005). Specifically, we used descriptive statistics to summarise study characteristics (e.g., year, location, and type of method) and thematic analysis to identify approaches and frameworks used to assess climate vulnerability in Africa.

**Results:**

We retrieved 94 articles in the review. Most studies were conducted in South Africa (14/94, 15%) and Ethiopia (16/94, 17%). Vulnerability assessments were predominantly conducted in the agriculture (29/94, 31%) and environmental science (30/94, 32%) sectors. Thirteen vulnerability frameworks emerged, with the majority associated with the Intergovernmental Panel on Climate Change assessment report four framework. The primary vulnerability methods were the use of linear aggregation (66/94, 70%) and unbalanced weighting (39/94, 41%). Flooding and rainfall were the most studied climatic hazard and EWEs in Africa. Few studies assessed climate vulnerability in health science, despite its critical importance.

**Conclusion:**

Existing frameworks demonstrate growing innovation; however, their methodological rigour varies, with inadequate contextual validation of indices and models. The methodological robustness enhances the selection of models that align with the specific dynamics and context of the system being evaluated. These methods guide policymaking and resource prioritization, by quantifying climatic vulnerability.

**Supplementary Information:**

The online version contains supplementary material available at 10.1186/s44329-025-00041-7.

## Background

Climate change has adversely impacted the global population through the destruction of infrastructure, compromised food production, and reduced water availability, leading to adverse health and well-being outcomes [1]. The frequency, magnitude, and extent of climatic hazards, exacerbated by climate change, have recently substantially increased [1, 2], posing a threat to the future of already vulnerable nations in the Global South. These have consequently become a hindrance to achieving Global Goals, the universal call to action to end poverty, protect the planet, and ensure that by 2030, all people are prosperous and live in peace [1]. Climate change is expected to accelerate impacts on both natural and human systems, and widen existing regional development disparities [3]. 

Climate vulnerability, the predisposition of a population to suffer negative impacts from hazardous climate events,[1] has been recognised as a crucial research priority for adaptation planning by the United Nations Environmental Programme [4]. Africa experiences significant climatic variability, and is highly vulnerable to climate change such as changes in precipitation, rising temperatures, and sea-level rise [1, 5, 6]. These changes threaten development, impacting key sectors and socio-economic welfare [5, 7]. Additionally, factors such as poverty, land degradation, and low adaptive capacity further heighten vulnerability [8]. Without adequate climate resilience measures, an estimated 118 million already impoverished individuals will face increased risks from floods, droughts, and extreme heat by 2030 [9]. Therefore, assessing population vulnerability is crucial to understanding Africa’s climate challenges.

Population vulnerability varies over time, and across different regions [10]. Some regions and demographic groups are particularly susceptible to the severe effects of climate hazards and EWEs [11]. Additionally, vulnerability can vary depending on the risk perspective employed, necessitating the use of distinct metrics. For example, social vulnerability highlights the characteristics and situations within social groups that heighten their susceptibility to adverse effects, [12] while health vulnerability focuses on the likelihood of experiencing health issues [13]. The variable nature of vulnerability requires a deeper understanding of how EWEs lead to disproportionate impacts [11]. This knowledge can be more effectively used to inform resource allocation and shape policy and decision-making [14]. Given that vulnerability is not always directly observable, a robust criterion for estimating and modelling vulnerability is essential. The complexities of vulnerability concepts and their diverse applicability necessitate a synthesis of information to develop country-specific frameworks [15].

Previous research synthesizing information on vulnerability assessment methods are limited. A global review by Pradyumma et al. (2022) indicated that most assessments were conducted in high-income countries [16]. The review focused on health vulnerability and presented a summary of frameworks and methods as integrated concepts in vulnerability assessment. Furthermore, while this was a global review, only one article from Africa met the inclusion criteria [16]. A review by Membele et al. [17] in the same year focused on flood vulnerability only in developing countries. The authors highlighted multi-criteria analytical approaches and Geographic Information Systems (GIS) as the most commonly used methods in assessing flooding vulnerability [17]. These studies enhanced the understanding of methods used to assess vulnerability due to climate change; however, they were constrained to specific disciplines and were global in scope, lacking a focus on the African region, which experiences some of the greatest impacts of climate change.

Consequently, a comprehensive and systematic review of the current state of vulnerability assessment across various disciplines is necessary. This is essential, given that vulnerability assessment transcends multiple disciplines in research, policy and across multiple scales. A regionally focussed review will highlight evidence on climate vulnerability assessment, identify knowledge gaps and provide regional perspective to inform research agendas, global discussions on vulnerability assessment and climate adaptation specific to the continent. We undertook this scoping review with a focus on Africa, using an updated search to enable a more regionally relevant understanding of approaches to climate vulnerability across sectors. The aim was to identify methodological approaches employed to assess climate vulnerability and needs for future research on vulnerability assessment related to climate change.

## Methods

We conducted the scoping review following the methodology established by Arksey and O’Malley [18]. The five main steps of the method were: identification of the research question; identification of relevant studies; study selection; data charting; and collating, summarizing and reporting the results. The scoping review protocol was registered in the Open Science Framework directory [19]. 

### Identification of the research question

The primary research question was, “What methods are employed to evaluate climate vulnerability in Africa?”

Specifically, we aimed to address the following questions:

1. What evidence is available regarding climate vulnerability assessment methods across Africa?
2. What are the vulnerability frameworks utilized in climate vulnerability assessment?
3. Which weighting methods are predominantly utilized in climate vulnerability assessment?

### Eligibility criteria

We used the population, concept, and context (PCC) framework to define the inclusion and exclusion criteria before carrying out the search. Our inclusion criteria were articles on Africa that used quantitative methods, focused on specific extreme weather events or climatic hazards, and described methods for calculating vulnerability indices. We limited the search to English-language articles published between 2003 and 2023 to capture patterns, advances, and shifts in knowledge over the past two decades.

We excluded articles conducted outside Africa, qualitative studies, articles without information on specific extreme weather or climatic hazards, studies on spatiotemporal climate variability, and those focused solely on future climate projections (Supplementary file 1, Appendix I).

### Search strategy

We sought published literature from peer-reviewed article databases and targeted grey literature sources. We searched academic databases, PubMed, Ovid Embase, Ovid Medline, Web of Science, EBSCOhost Medline, and CINAHL Complete. For the grey literature, we included relevant articles from the United Nations (UN) [20] and the United States Agency for International Development (USAID) [21] selected for their direct focus on climate vulnerability in Africa, methodological rigour, and alignment with our inclusion criteria. Additional literature was identified by screening the reference list of identified eligible studies.

We conducted the search using the abstract, title, and keywords across all databases. We constructed the search strings in alignment with the research questions, developed through collaborative consultation and discussion among the coauthors. This process involved iterative refinement to ensure the search terms comprehensively capture the study scope. We initially identified key concepts and synonyms in relation to the research question. We then piloted the search strings across multiple databases and compared the preliminary findings against a set of known relevant articles to confirm that the strategy could reliably retrieve them. Feedback from all coauthors was incorporated to refine the wording and reduce irrelevant retrieval. Through this process we ensured the final search strings were comprehensive, and transparent to capture a wide range of possibly relevant studies.

The search strategy required that articles include at least one keyword from the specified topics (climate change, climate, climate risk, flood, extreme weather, extreme temperature, extreme rainfall, drought, and cyclone), vulnerability terms (vulnerability, disaster, and susceptibility), and region-specific keywords on various African nations. The search included climate, vulnerability, Africa, and country names within Africa as keywords and then combined them using Boolean operators “OR” and “AND” to ensure the inclusion of all suitable publications. Details of keywords and combinations highlighting the search strategy from all databases are presented in the supplementary file 1, Appendix II. Grey literature was included based on a search of the keywords, “climate”, “vulnerability”, and “Africa” to identify reports focused on our research question. We then imported all identified articles into Covidence software [22] for screening based on eligibility criteria and data extraction.

### Article selection

The review followed the guidelines of the Preferred Reporting Items for Systematic Reviews and Meta-analyses extension for Scoping Reviews (PRISMA-ScR) checklist [23]. The checklist is available in supplementary file 2. We imported all articles into the Covidence Software and automatically de-duplicated for further review. Title and abstract screening were conducted by five independent reviewers (EO, SAO, EAO, EK, MCK) against the inclusion and exclusion criteria. A sample of the articles included (10%) was then reviewed for systematic error by a second reviewer (MCK). Two independent reviewers (EO and SAO) then fully double-reviewed the full text of all relevant articles. We evaluated the inter-rater reliability to assess consistency between the two reviewers, (Cohen’s kappa = 0.88) and the agreement was substantial. In the event of a conflict in decision or disagreement, a third reviewer (MCK) was consulted to reach a final decision, and any ineligible articles were excluded with reasons documented.

### Data extraction

Data extraction was performed using a pre-defined and tested template uploaded to the Covidence software. Two independent reviewers (EO and SAO) carried out the data extraction, and any disagreements were resolved through discussions between the two reviewers and a third investigator (MCK). The information extracted for each article included: (1) bibliography; (2) vulnerability frameworks; (3) vulnerability models; (4) indicators; (5) vulnerability dimensions; (6) types of indices investigated; (7) weighting methods; and (8) climatic hazards and extreme weather events covered. Vulnerability frameworks entailed organised conceptual approaches of vulnerability assessment whereas vulnerability models were the methodologies utilised to come up with a vulnerability index. Vulnerability models were classified by the equation structure and the final function, categorised into factors, generic domains, or climatic domains. Vulnerability domains are broad categories or dimensions that group related factors. Factor-based models assessed individual indicators such as income level, education, or health status; climatic domains focus on exposure, sensitivity, and adaptation; while generic domains encompass various groupings such as social, economic, and environmental. Indicators included the numerous covariates included in the vulnerability assessment, while the type of indices investigated were the specific vulnerability computed, such as health vulnerability.

### Data analysis and presentation

We provided a summary of the study’s characteristics, models for evaluating vulnerability, and the various domains used to categorise vulnerability traits into broad, distinct categories. We extracted the data into subcategories based on content, as outlined in the previous section and displayed in a detailed results table. The contents of this table underwent further descriptive analysis using frequencies and percentages and were visually summarised following the scoping review guidelines. A narrative summary accompanies both the tabular and visual representations.

### Role of the funding source

The funders of the study had no role in the study design and conduct, data collection, data management, data analysis, data interpretation, or writing of the manuscript.

## Results

### Included studies

**Fig. 1:**
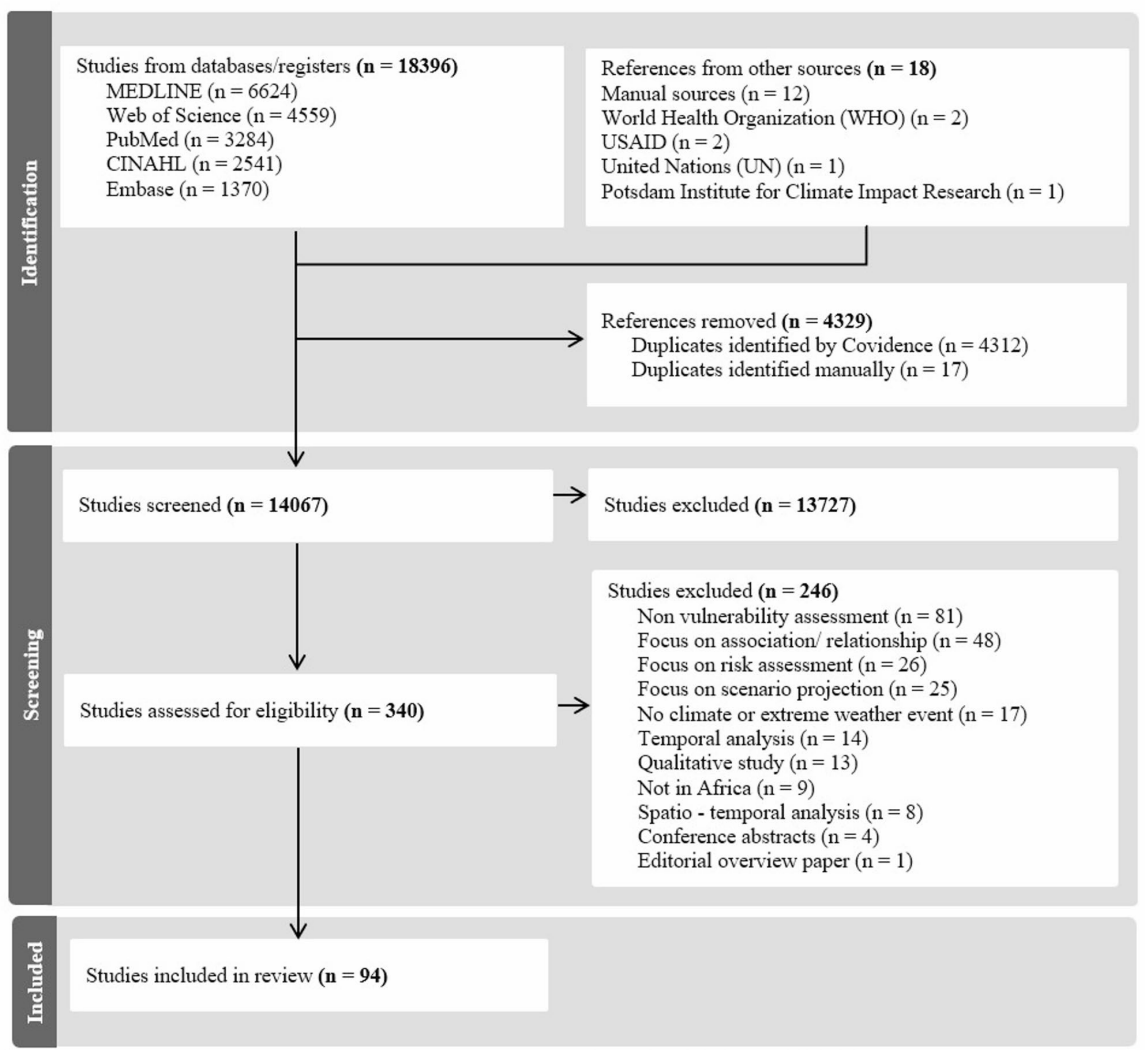
PRISMA Flowchart

We identified 18,396 articles based on an initial literature search. We excluded (4329/18396, 24%) articles as duplicates across searched databases, and (14067/18396, 76%) articles were subject to title and abstract screening based on the inclusion and exclusion criteria (Supplementary file 1, Appendix I). Out of a total of (340/18396, 2%) retained for full-text review, 246 full-test articles were further excluded based on the inclusion criteria. Finally, 94 articles were included for data charting and synthesis (Fig. 1).

### Features of the included studies

**Fig. 2:**
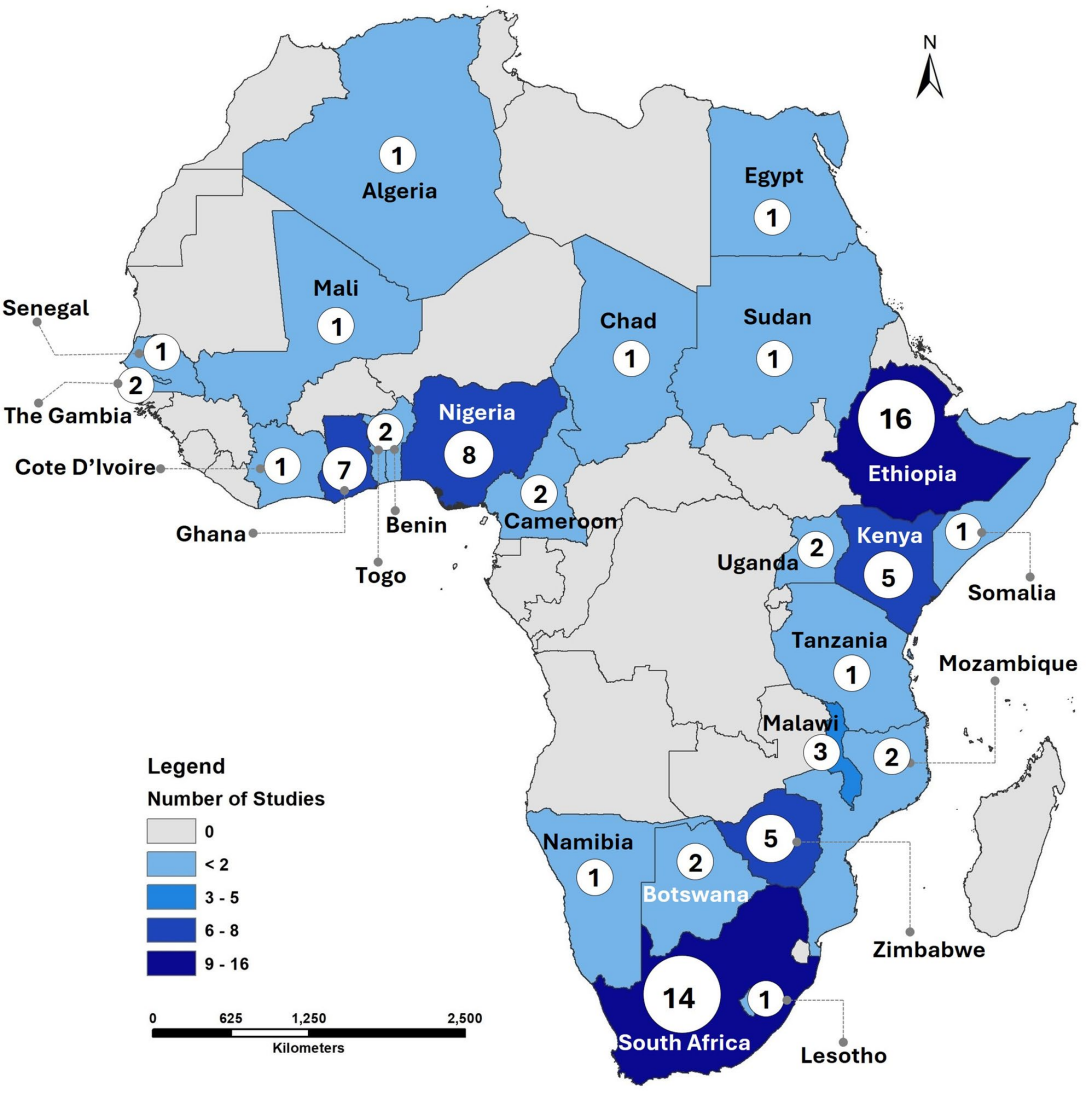
Spatial distribution of articles reviewed in various countries across in Africa

Ethiopia (16) and South Africa (14) had the highest number of studies, of the 94 articles included. Single studies were identified from eleven countries (Fig. 2). Four studies addressed the entire African continent, whereas two focused on Sub-Saharan Africa (SSA) and West Africa each. One global article was also included because it assessed vulnerability inclusive of Africa (Supplementary file 1, appendix III).

**Fig. 3:**
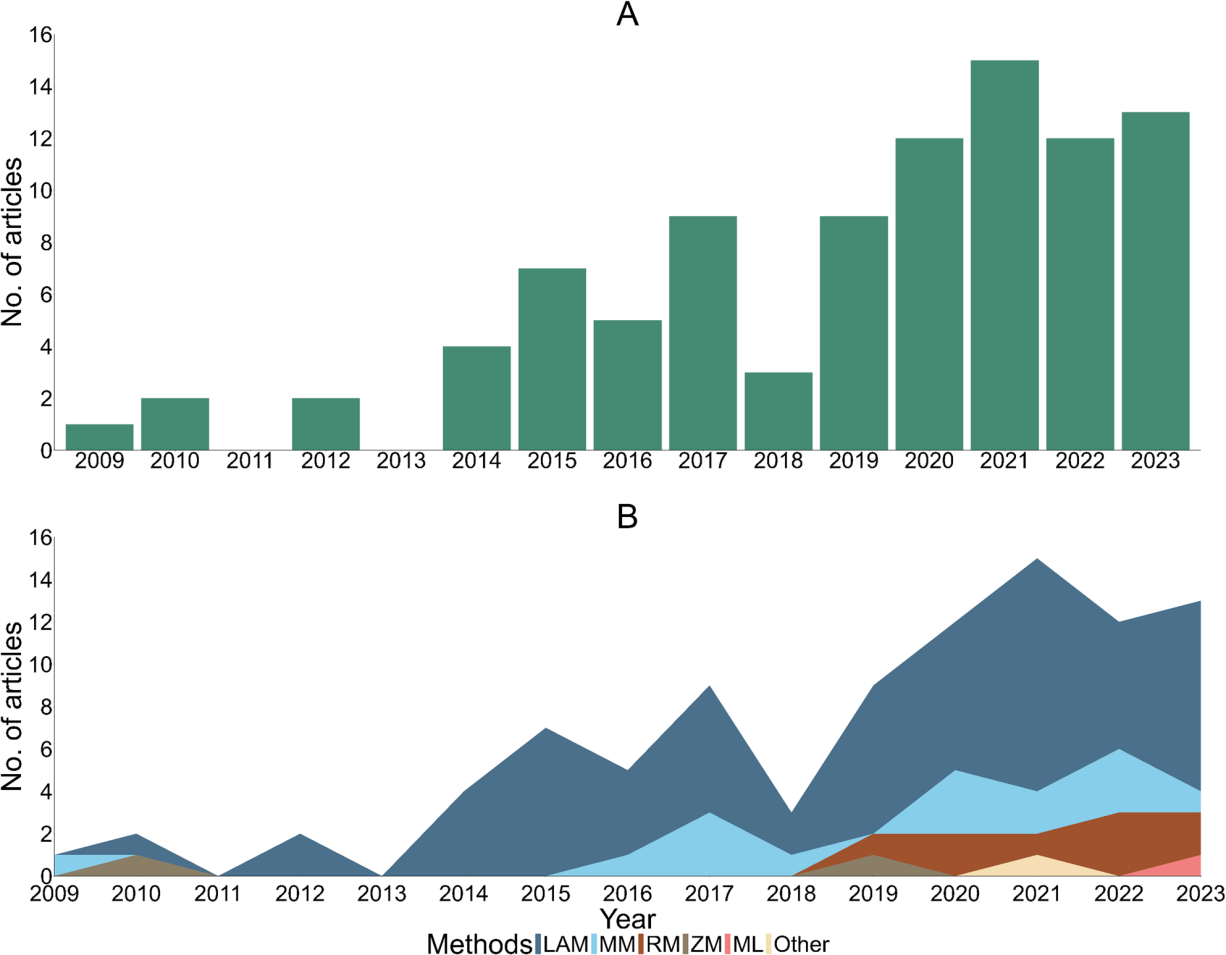
Panel **A**- Summary of articles included in the review by year of publication, Panel **B** – Vulnerability models by year of publication. Panel **B**: Linear aggregation model (LAM), Multiplicative Model (MM), Ratio Model (RM), Zscore-based Model (ZM), Machine Learning Model (ML), Other. The y-axis represents the number of articles reviewed while the x-axis represents the year of publication. Although the review was between 2003 and 2023, no article was included prior to 2009, in 2011 and 2013

The number of vulnerability assessment publications has increased steadily over time (Fig. 3 – Panel A). The first published study was identified in 2009, and the number of articles increased steadily to 13 by 2023. The highest number of articles (*n* = 15) was reported in 2021, while there were no studies reported in 2011 and 2013 (Fig. 3).

**Fig. 4:**
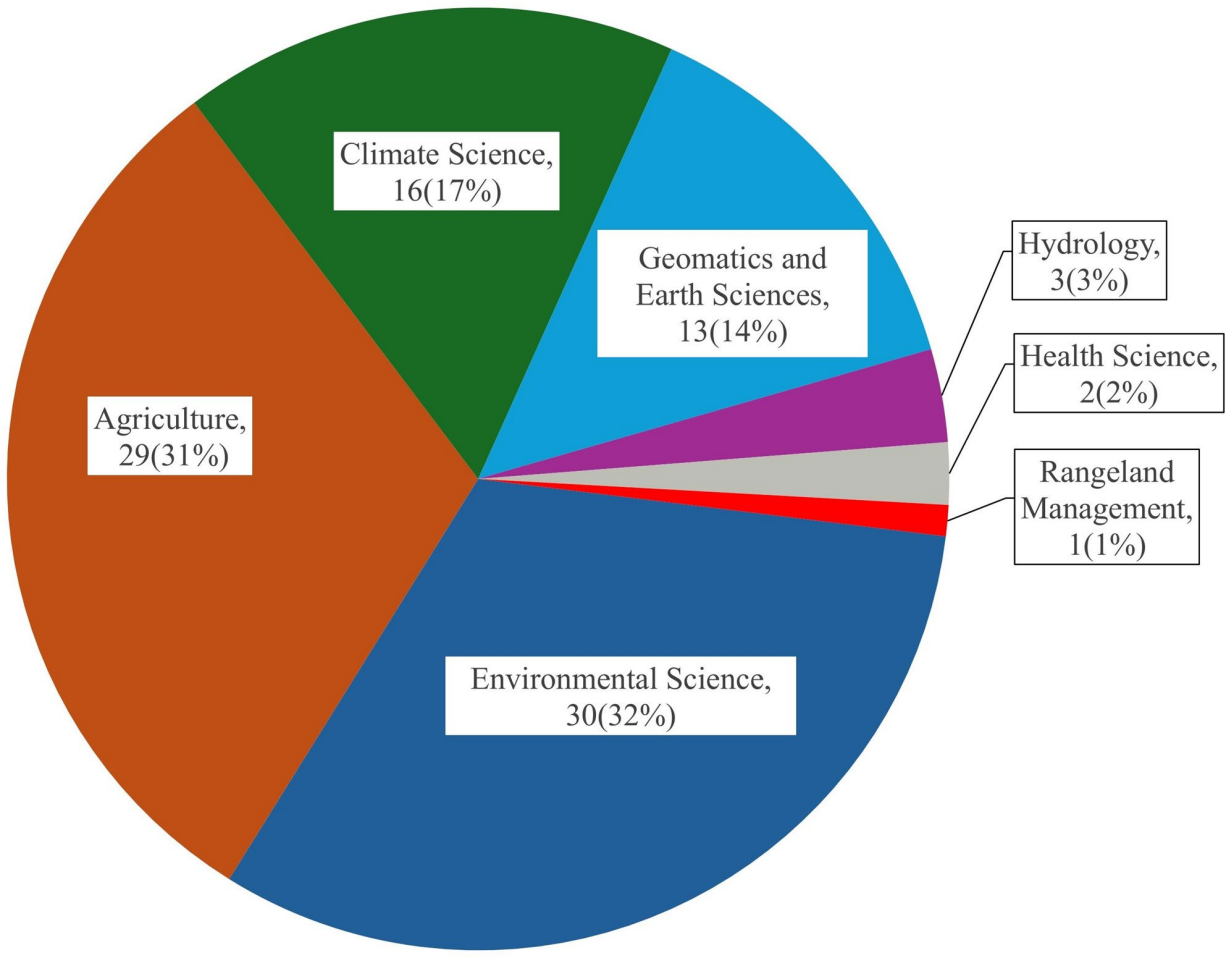
Subject disciplines covered. Categorization of articles based on the various fields of study covered in the review

The field of study varied across various disciplines, ranging from one study of rangeland management to (30/94, 32%) in environmental science and (29/94, 31%) in agricultural sector. In addition, only two articles from health sciences were retrieved (Fig. 4).

### Frameworks used to define vulnerability

We identified 13 frameworks that had been applied to calculate vulnerability indices (Table 1). The majority of the studies, (35/54, 65%), used the Intergovernmental Panel on Climate Change (IPCC) frameworks to categorise the indicators. Categorisation of the indicators based on the IPCC framework varied based on the specific assessment report (AR) referenced. Among the 35 studies, using the IPCC framework, (31/35, 89%), were associated with IPCC assessment report four (AR4), categorising the indicators into exposure, sensitivity, and adaptive capacity, while only one study was associated with IPCC’s AR3.

**Table 1:** Summary of vulnerability frameworks, models and weighting approaches identified in the review

Name			Description	Number of papers	Citation
**Vulnerability Frameworks**					
Intergovernmental Panel on Climate Change	Assessment Report 3		Provides scientific guidance to the UNFCCC and Paris Agreement, aiding in climate policy development, vulnerability identification, awareness, and monitoring. Expresses vulnerability as function of exposure, sensitivity and adaptive capacity.	1	[24]
	Assessment Report 4		Expresses vulnerability as function of exposure, sensitivity and adaptive capacity.	31	[13, 20, 21, 25–52]
	Assessment Report 5		Introduced the concept of vulnerability as a function of risk. Vulnerability is expressed as a function of sensitivity and adaptive capacity.	3	[53–55]
Bogardi, Birkman, Cardona Framework			Addresses the complexity of vulnerability and resilience to social, ecological, and economic shocks. Uses exposure, susceptibility, and coping capacity as key dimensions	5	[12, 56–59]
Method for the Improvement of Vulnerability in Europe Framework			Provides a comprehensive approach to assessing key factors and various dimensions of vulnerability to human-induced and natural hazards. Vulnerability is expressed as function of exposure, susceptibility, and lack of resilience.	3	[60–62]
Vulnerability Sourcebook Framework Adopted from Fritzsche et al. (2014)			Provides a standardized approach to the assessment of both climate vulnerability and risk to be used for adaptation planning. Expresses vulnerability as a function of exposure, sensitivity and adaptive capacity.	2	[63, 64]
Vulnerability Framework of Turner et al. 2003			Identifies and measures risks and vulnerability before and after a disaster occurrence, emphasizing the interactions between human and environmental systems.	2	[65, 66]
Vulnerability to Resilience Framework Developed by Practical Action in 2011			Assesses vulnerability to hazards and stresses in communities, using a community-centered approach, strengthening their resilience to climate change impacts.	1	[67]
Sustainable Livelihood Framework			Presents the main factors affecting people’s livelihoods and assets, and the typical relationships between these factors.	1	[68]
Landscape Vulnerability Framework			Assesses the impact of climate change and other factors on landscapes by identifying most vulnerable areas and inform conservation and adaptation strategies. Used exposure, sensitivity and adaptive capacity as key dimensions.	1	[69]
West Sudanian Savannah Vulnerability Framework			Assesses vulnerability to environmental and human-induced factors through the interaction of socio-economic, ecological, and climatic factors, to improve community resilience.	1	[70]
Social Ecological Technological Framework			A conceptual model that examines the connections between social, ecological, and technological systems, emphasizing human-environment interactions.	1	[71]
Hyogo Framework for Action (Birkmann, 2006)			Considers vulnerability assessment in the form physical, social, economic and environmental characteristics and conditions.	1	[72]
**Vulnerability Models**					
Linear aggregation			Combination of multiple variables using a linear function that includes, summing, averages and weighted averages.	66	[12, 20, 21, 24–26, 28, 42, 44, 45, 47–60, 63, 66–68, 70–107]
Multiplicative model			Combines the values of a set of indicators into a single aggregate using a product function.	15	[13, 29–41, 61]
Ratio model			Arithmetic models that create a composite index based on division of variables or dimensions.	9	[43, 46, 62, 64, 65, 69, 108–110]
Zscore based model			Standard score that estimates the deviation of data points from the mean.	2	[111, 112]
Machine learning approaches (random forest, K- nearest neighbors, support vector machine, and naive bayes)			Involves using algorithms to analyze large datasets and predict areas or populations at risk based on patterns and trends in historical and real-time data.	1	[27]
Other			Adopted a vulnerability index developed by Food Agriculture and Natural Resources Policy Analysis Network (FANRPAN).	1	[113]
**Weighting Methods**					
Balanced/equal			Provides equal level of importance to all indicators.	32	[12, 13, 21, 25, 29–33, 35–38, 40, 42, 46–48, 53, 56–58, 64, 67, 72, 73, 85, 89, 91, 100, 101, 104]
Unbalanced/unequal		PCA	Utilizes the explained variance to determine the weights assigned to the principal components.	13	[39, 49, 52, 54, 55, 63, 74, 77, 78, 96, 97, 99, 112]
		MCDA	Method that explicitly evaluates multiple conflicting criteria in decision.	12	[71, 76, 79–84, 86–88, 95]
		Expert judgement	Experts are involved in the prioritization of the indicators based on the level of importance.	5	[44, 59, 70, 75, 105]
		Lyengar and Sudarshan	Assumes weights to vary inversely of variance.	4	[24, 61, 66, 93]
		Respondent perception	Considers respondents perception on the level of importance on the various indicators.	2	[94, 113]
		Variation coefficient used as weights	Uses coefficient of variation as the weights	1	[102]
		Simple proportions approach	Weights assigned based on the fraction of the total number of variables.	1	[51]
		Other	Indicators weighted based on the level of importance, but the specific unbalanced weighting methods was not provided.	2	[106]

Table 1 summarizes vulnerability frameworks identified in the review, categorization of the frameworks is based on vulnerability dimensions: exposure, sensitivity and adaptive capacity. Abbreviations: Multi criteria decision analysis (MCDA), Principal component analysis (PCA). MCDA includes AHP, ANP, fuzzy logic and those considered as MCDA in general

The Methods for Improvement of Vulnerability in Europe (MOVE) framework and the Bogardi, Birkman, and Cardona (BBC) Framework were utilised in three and five studies, respectively. Only one article used the Vulnerability to Resilience Framework developed by Practical Action in 2011, the Sustainable Livelihood Framework, the Landscape Vulnerability Framework, the West Sudanian Savannah Vulnerability Framework, the Hyogo Framework for Action and the Social Ecological Technological Framework. A detailed description of the frameworks is provided in Table 1.

### Models used to characterise vulnerability

There were various statistical models that were used within the frameworks to combine indicators and or sub-domains to create overall indices or sub-domain indices. Table 1 summarises these models, and further details are presented in the supplementary file 1 (Appendix IV and V). Overall, six models were identified from the 94 studies. Majority (66/94, 70%) of the articles utilized a linear summation model to calculate vulnerability. That is, the scaled values of indicators of sub-domains were summed through a linear summation to create an index. Multiplicative models were utilized in (15/94, 16%) articles where the model combined a set of indicators using a product function. Machine learning (ML) approaches were identified in one article. Among the specific ML approaches analysed were random forest, K-nearest neighbours, support vector machines, and naive Bayes.

These models either considered individual factors or grouped the factors into sub-domains (dimensions) before inclusion into the main model. There were two categories of domains: climatic and generic domains. The sub-domains within the climatic category (55/94, 59%) were exposure, sensitivity and adaptive capacity, while the generic category (16/94, 17%) contained sub-domains such as demographic, economic and social factors. A detailed summary of these categories and corresponding sub-domains is provided in supplementary file 1, appendix V.

The trend in vulnerability models highlights that linear aggregation increased steadily across the years, being least used in 2010 (*n* = 1) and most used in 2021 (*n* = 11). Multiplicative models were adopted over the years, reaching the highest utilisation in three articles in 2017, 2020 and 2022, respectively. In recent years, other models have been adopted, including machine learning approaches (*n* = 1) in 2023 (Fig. 3 – Panel B).

### Analysis of weighting methods in vulnerability assessment

Weighting methods were grouped into balanced/equal (32/94, 34%); unbalanced/unequal (39/94, 41%), and no weighting (23/94, 24%). There were eight unequal weighting schemes with Principal Component Analysis (PCA) (13/39, 32%) and Multi criteria decision analysis (MCDA) (12/39, 29%) as the most utilized, while variation coefficient and simple proportions utilized in only one article (Table 1). The MCDA methods highlighted included the analytical Hierarchy Process (AHP), the Analytic Network Process (ANP), and fuzzy-based approaches.

### Types of vulnerability indices

The articles examined various vulnerability indices, such as the livelihood vulnerability index, flood vulnerability index, and health vulnerability index as shown in supplementary file 1, appendix VI. Flood vulnerability at (26/94, 28%), livelihood vulnerability at (15/94, 16%), and social vulnerability at (14/94, 15%) were the most assessed indices.

### Overview of vulnerability indicators

The studies incorporated a mixed set of indicators in vulnerability assessment, which varied across studies. The primary grouping of indicators considered was exposure, sensitivity and adaptive capacity. In some articles, this was further grouped into sub-components, such as exposure, natural hazards and climate variability, sensitivity, health security, water security, food security, adaptive capacity, livelihood strategies, socio-demographic profile, and social networks, in a livelihood vulnerability index. Other domains outside the IPCC key climatic domains included ecological, economic, infrastructure, and social aspects in a socio-ecological vulnerability index (Supplementary file 1, Appendix VI).

The included studies covered a range of climatic hazards and extreme weather events, including flooding, drought, cyclones, extreme temperatures, precipitation, sea level rise, storms, earthquakes, coastal erosion, and strong winds. The most studied climatic hazards and extreme weather events were flooding and rainfall, (76/143, 53%), while the least were earthquakes and strong winds, respectively one article (Supplementary file 1, appendix VII).

## Discussion

Our scoping review of methodologies for defining vulnerability yielded 94 articles published in Africa over two decades, between 2003 and 2023. Over half of the reviewed studies were conducted in 4 of the 25 countries where vulnerability methods were described. The measurement of climate vulnerability over the 2 decades incorporated a wide range of fields; however, environmental science and agriculture accounted for over 60% of all the published articles. Comprehending the specific vulnerabilities of the population to climate challenges is essential for effective response strategies. By assessing population vulnerability and exposure to climate change impacts, we can prioritize actions for those most at risk, thereby improving the living environment for the population. As climate impacts intensify, science-driven insights are crucial for enhancing resilience and informing adaptation strategies that support sustainable development.

From our review, while there was heterogeneity in the methodological vulnerability frameworks, there were broad similarities in categorizing indicators into three domains of exposure, sensitivity, and adaptive capability, following the IPCC framework. The significance and increased interest in measuring climate vulnerability is shown by a consistent rise in publications since 2003. These coincide with a notable rise in funding for climate and research initiatives [114, 115]. Similarly, the use of vulnerability methods has progressed, primarily using a linear summation approach and various weighting techniques.

The predominance of vulnerability assessments in agriculture and environmental sciences likely reflects SSA’s heavy reliance on rain-fed agriculture, which supports the livelihoods and food security of millions [25]. Climate change, leading to erratic rainfall, and prolonged droughts, threatens crop yields and exacerbates vulnerability, likely driving the focus on this sector to identify at-risk areas and adaptation options. While this emphasis is critical, it may overshadow other agricultural challenges, such as soil degradation or shifts in crop suitability, and risk neglecting diverse systems like pastoralism. Environmental assessments complement this focus, addressing vulnerabilities such as urban flooding caused by impervious surfaces [26], deforestation, and climate variability across regions [9]. These environmental studies enhance disaster preparedness and highlight adjustments for sustainable development, such as improved drainage or land-use planning. However, our review suggests a need for broader assessments to capture urban-rural dynamics and long-term climate impacts, ensuring a more comprehensive approach to vulnerability in Africa.

While agriculture and environmental sciences dominated our review, only two studies, less than 5% of the total, addressed health sciences, revealing a striking gap in vulnerability assessments. This is concerning, as climate change exacerbates health risks, including spread of malaria, heat-related illnesses, and malnutrition, particularly in SSA [5]. The limited focus may stem from challenges such as scarce health data, funding biases toward agriculture, or the complexity of measuring the climate’s impacts on nutrition and disease. This underrepresentation risks skewing adaptation strategies, leaving policymakers ill-equipped to tackle interconnected vulnerabilities in health and other sectors. Our review highlights the pressing need for integrated frameworks that combine agriculture, environment, and health thereby guiding future research to address Africa’s complex climate challenges more wholistically.

Diverse vulnerability frameworks were identified from the review, with the IPCC frameworks particularly AR4, predominating in 31 of 35 studies (89%) due to their structured approach in categorizing indicators into exposure, sensitivity, and adaptive capacity. While these domains provide a consistent foundation for assessing vulnerability, their universal application may overlook Africa-specific challenges, such as informal economies, conflict, or limited data availability, which are critical to understanding regional vulnerability. The prevalence of AR4, established in 2007, likely reflects its familiarity and ability to produce separate vulnerability maps, aiding decision-makers in targeting adaptation options [21, 27, 28]. However, the slow uptake of AR5 (2014), which frames vulnerability within a broader risk assessment, raises concerns. This lag may stem from practical barriers, such as limited training or data, or a perception that AR4 better suits African contexts, although these risks oversimplify complex socio-ecological systems [116].

Non-IPCC frameworks, such as MOVE, BBC, and the Sustainable Livelihood Framework, were less common and were applied in only three to five studies each. This underuse may reflect their complexity or lack of tailoring to African settings; yet their focus on social, ecological, or technological dimensions could complement IPCC approaches. Our review highlights a critical gap: the need for frameworks that integrate local perspectives and address Africa’s unique vulnerabilities. By mapping the dominance of AR4 and the diversity of alternatives, this study highlights the urgency of developing context-specific tools to enhance climate vulnerability assessments, guiding researchers and policymakers toward more effective adaptation strategies in Africa. The IPCC frameworks are widely utilised globally due to their significant influence on the development of international standards for climate change adaptation, including guidelines on vulnerability, impacts, and risk assessment [116]. There is a necessity for more localised and innovative approaches to vulnerability assessment. Future vulnerability frameworks should enhance inclusivity to effectively capture Africa’s distinct vulnerabilities in climate resilience planning.

Several models were identified from the review which help to statistically combine the indicators or sub-domains into an index, each with distinct implications for measurement accuracy and interpretability. Most of these articles applied a simple linear summation of indicators due to the simplicity in computation and ease of result interpretation, which has not been widely explored in Africa for vulnerability assessment. Linear summation models effectively capture overall magnitude and maintain comparability without distorting the relationship among indicators or domains. However, they lack compensation for extreme values and, due to their additive nature, may fail to account for interactions between indicators, reducing the model’s ability to reflect complex relationships within vulnerability assessments. In comparison, multiplicative models introduce an exponential growth to the index and may distort index values if one component is significantly larger or smaller. They also account for relationships among indicators, ensuring that poor performance in one area substantially impacts the overall index. While this can effectively highlight extreme conditions, it may also overinflate or suppress vulnerability scores, leading to misleading interpretations. Despite these concerns, frameworks such as the Livelihood Vulnerability Index, developed by the IPCC, leverage multiplicative models to emphasize compounding risks [13, 29–41]. While ratio models introduce a proportional relationship among the indicators or domains, they may inherently impose an inverse relationship, suggesting that improvement in the denominator lowers the vulnerability. In addition, machine learning techniques utilised in a single article are recognised for enhancing vulnerability index by managing complex interactions among various indicators and improving predictive accuracy through advanced algorithms, which have not been widely explored in Africa for vulnerability assessment.

Despite differences in vulnerability assessment models, Iliyyan et al., while comparing three index methods and applying the same data to the assessment, found that the vulnerability scores still fell within the same categories, likely leading to similar results [117]. This suggests that, while methodological variations exist, overarching trends in vulnerability assessment tend to converge. However, this does not inherently imply methodological robustness; instead, it highlights the need for contextual validation of index selection, ensuring that the selected model aligns with the specific dynamics of the system evaluated. Our review emphasises that understanding the trade-offs of each approach is essential for selecting a model that effectively represents vulnerability dynamics while maintaining interpretability and analytical accuracy.

The temporal scope of the vulnerability models revealed a progressive increase in vulnerability assessment over time, accompanied by the emergence of newer methods, including machine learning approaches and artificial intelligence. These methods have proved valuable as they can use increasing amount of data and can predict future vulnerabilities based on historical data and emerging trends [118]. This transition indicates a trend toward adaptive and data-driven evaluations, complementing traditional methods with more dynamic, yet computationally demanding, techniques. Despite the emergence of new methods, the persistent significance of traditional models underscores their continued relevance in vulnerability assessments, particularly in contexts where computational resources are limited.

The emphasis on weighting methods in various articles ensures that vulnerability indices are contextually relevant, statistically robust, and practically applicable for policymaking. Equal weights were considered mainly due to their simplicity and the assumption that all indicators contributed equally to vulnerability [42]. In some cases, equal weights were applied due to a lack of empirical rationale for assigning differential weighting to the indicators [25, 73]. In addition to the equal weighting scheme, various statistical and expert-driven approaches have been employed to refine the allocation of indicator weights. Most studies applied either PCA or MCDA. Although considered objective and computationally feasible, PCA [74] minimizes the contribution of individual indicators [75]. Therefore, PCA should be preferred in the construction of indices in cases where well-defined weights are absent to ensure a data-driven approach [63] or where the number of indicators included is numerous, necessitating data reduction. MCDA has gained traction as a semi-quantitative approach that combines qualitative approaches, such as expert and respondent opinions, with a form of ranking [119]. Qualitative and semi-quantitative approaches are widely utilised due to their simplicity, ability to manage data scarcity, and have proved useful in regional studies [119]. Although commonly used among the MCDA methods, AHP has been criticised for its simplicity, as it assumes that certain criteria are entirely independent and does not accommodate multiple alternatives simultaneously [17]. Alternative MCDA approaches, such as ANP or fuzzy-based methodologies, are considered to address some of these challenges [17, 76]. 

Expert opinion as a weighting approach remains advantageous, especially in policy-driven reflection, where weighting should align with government priorities [70]. The approach, however, is limited when a consensus on the weights cannot be reached among expert panel members [75]. This has been addressed by incorporating local community knowledge and traditional values in the weighting process [75] to ensure vulnerability models reflect ground realities rather than theoretical assumptions. The integration of contextual validation, hybrid methodologies, and local expertise enhances the effectiveness, adaptability, and responsiveness of weighting methods to real-world vulnerabilities. This is advantageous as it enhances relevance, provides an indigenous perspective, and ultimately strengthens ownership of the findings while encouraging proactive adaptation.

Africa is facing escalating climate hazards, with extreme weather events including droughts, heavy rainfall, floods, and heatwaves becoming more frequent and severe [9]. In this review, the most studied climatic indicators were floods, rainfall, droughts, and temperature. By emphasising these climatic events, it underscores the continent’s vulnerability across different sectors and the urgent need for actions to bolster resilience. Responding to climate change is becoming increasingly crucial, and recognising these key indicators from this review requires improved commitment to adaptation and mitigation strategies at both the regional and national levels. To provide a comprehensive understanding of climatic hazards, the IPCC AR5 framework integrates climatic hazards in a holistic risk assessment, providing a multidimensional view that allows for proactive adaptation and reduction of adverse impacts [120]. Governments and policymakers need to prioritise climate-resilient infrastructure, enhance early warning systems, and reinforce community adaptation initiatives to mitigate climate vulnerability effectively.

This review highlights significant deficiencies in climate vulnerability assessments throughout Africa. Some methodologies have neglected the dynamics between urban and rural areas, and the interconnected vulnerabilities that affect health, agriculture, and the environment. Existing frameworks exhibit increasing innovation; however, their methodological robustness is inconsistent, with insufficient contextual validation of indices and models. In the absence of such validation, tools may inadequately represent the diverse climate realities of Africa. Future research must emphasise localised, contextually relevant methodologies and integrated cross-sectoral frameworks that address systemic vulnerabilities. Addressing these priorities will enable future research to develop comprehensive, innovative, and practical vulnerability assessments that strengthen climate resilience in Africa while also improving the ownership, and policy relevance of adaptation planning.

### Strengths and limitations of this study

To our knowledge, this is the first comprehensive review to examine vulnerability to climate change and extreme weather events across multiple disciplines in Africa covering two decades (2003–2023) of evidence. The review unpacks a range of methods that can be applied in future vulnerability assessments, adaptable to specific fields of study, local areas, regions, or countries. This scoping review must be considered in light of certain limitations. This review focused mainly on quantitative methods for assessing vulnerability. Nevertheless, qualitative approaches have also been used to evaluate vulnerability [121–123] and may often uncover narratives that are frequently overlooked in particular contexts of vulnerability such as social and cultural drivers. Studies from non-English speaking regions, may have been overlooked since the review was restricted to papers published in English potentially overlooking valuable regional and local knowledge in the specific regions.

## Conclusion

Assessing climate change vulnerability in Africa is vital for identifying at-risk communities and guiding interventions. This review outlines methods for estimating vulnerability, applicable to developing indices, with choices depending on frameworks, weighting, and specific approaches. Although the IPCC AR5 framework is more recent, the IPCC AR4 framework dominated climate vulnerability assessments within our study period. Most studies were concentrated within agriculture and environmental sciences, with limited attention to health, highlighting an important research gap in the literature.

Over time, there has been a progressive increase in climate vulnerability assessments, accompanied by the adoption of newer vulnerability models. Furthermore, the integration of expert opinion and community knowledge has emerged as a key approach to ensure that vulnerability models more accurately reflect ground realities and provide contextually relevant insights. These methodological advancements not only enhance the reliability of assessments but also improve their utility in various policy and planning contexts.

These methods inform policymaking and resource prioritization, by quantifying climatic risks and enhancing adaptation measures, thereby facilitating evidence-based adaptation planning at local, national, and regional levels. However, vulnerability assessment remains complex and context dependent, requiring further empirical and theoretical refinement of approaches to validate their effectiveness. Using appropriate tools is crucial to ensure that interventions are not only technically sound but also socially inclusive and locally grounded.

## Supplementary Information

Below is the link to the electronic supplementary material.

Supplementary Material 1

Supplementary Material 2

## Data Availability

All data generated or analysed during this study are included in this published article and its supplementary information files.

## Abbreviations

EWEs: Extreme Weather Events
UNEP: United Nations Environmental Programme
GIS: Geographic Information System
PCC: Population Concept Context
UN: United Nations
USAID: United States Agency for International Development
PRISMA ScR: Preferred Reporting Items for Systematic Reviews and Meta-analyses extension for Scoping Review
IPCC: Intergovernmental Panel on Climate Change
AR: Assessment Report
MOVE: Methods for Improvement of Vulnerability in Europe
BBC: Bogardi, Birkman, and Cardona
HPM: Hazard of Place Model
ML: Machine Learning
BBN: Bayesian Belief Network
PCA: Principal Component Analysis
FAMD: Factor Analysis of Mixed Data
MCDA: Multi Criteria Decision Analysis
AHP: Analytical Hierarchical Process
ANP: Analytic Network Process
