# Supplementary material for: Methods for assessing climate vulnerability in Africa across two decades: a scoping review
Source: BMC Environ Sci. 2025 Dec 3;2(1):27. doi: 10.1186/s44329-025-00041-7 (PMC12684232; doi:10.1186/s44329-025-00041-7)
Supplement: Supplementary file 1 — Supplementary Material 1 [file 44329_2025_41_MOESM1_ESM.pdf]

Supplementary File 1:

Methods for Assessing Climate Vulnerability in Africa across two decades: A Scoping Review

List of Authors

Emily Odipo, Sharon A. Onyango, Moses C. Kiti, Robert W. Snow, Benjamin Tsofa, Jacob Mcknight, Peter M. Macharia, Emelda A. Okiro

Table of Contents

Appendix I: Inclusion and exclusion criteria for the scoping review 1

Appendix II: Search strategy 2

Appendix III: Regions/Countries covered in various articles 4

Appendix IV: Summary of vulnerability models 5

Appendix V: Vulnerability index models highlighted in the review 6

Appendix VI: Vulnerability index and the various major sub-component of the indicators 11

Appendix VII: Climatic hazards and extreme weather events 13

References 14

Appendices

Appendix I: Inclusion and exclusion criteria for the scoping review

Inclusion criteria	Exclusion criteria
Study area: Africa	Study area: not within Africa
Quantitative articles	Qualitative articles
Articles that identify an extreme weather event (e.g., drought, cyclone, extreme rainfall, extreme temperature) and an outcome influenced by the event through a vulnerability method.	No information about an extreme weather occurrence or focused on extreme weather event but not linked to an outcome
Articles that identify the methodology used to calculate a vulnerability index	Spatial-temporal climate variability and trends articles
Publication period: 2003 -2023 (20 years)	Scenario projection of climate change articles.
	Non-scientific articles (e.g., Commentry, Opinion)

Appendix II: Search strategy

PubMed:

((((("Climate change" OR climate OR "climate risk" OR flood* OR "extreme weather" OR "extreme temperature" OR "extreme rainfall" OR drought OR cyclone)) AND ((vulnerability OR disaster OR susceptibility))) AND ((Africa OR Algeria OR "São Tomé e Príncipe" OR Angola OR Benin OR Botswana OR "Burkina Faso" OR Burundi OR "Cape Verde" OR "Cabo Verde" OR Cameroon OR "Central African Republic" OR Chad OR Comoros OR Congo OR "Democratic Republic of Congo" OR "DRC " OR "Cote d'Ivoire" OR "Ivory Coast" OR Djibouti OR Egypt OR "Equatorial Guinea" OR Eritrea OR Eswatini OR Swaziland OR Ethiopia OR Gabon OR Gambia OR Ghana OR Guinea OR "Guinea-Bissau" OR Kenya OR Lesotho OR Liberia OR Libya OR Madagascar OR Malawi OR Mali OR Mauritania OR Mauritius OR Morocco OR Mozambique OR Namibia OR Niger OR Nigeria OR Rwanda OR "Sao Tome and Principe" OR Senegal OR Seychelles OR "Sierra Leone" OR Somalia OR (South* Africa) OR "South Sudan" OR Sudan OR Tanzania OR Togo OR Tunisia OR Uganda OR Zambia OR Zimbabwe))) AND (English[Language])) AND (("2003/01/01"[Date - Publication] : "2023/12/31"[Date - Publication]))

Ovid (Embase):

(("Climate change" OR climate OR "climate risk" OR flood* OR "extreme weather" OR "extreme temperature" OR "extreme rainfall" OR drought OR cyclone) and (vulnerability OR disaster OR susceptibility) and (Africa OR Algeria OR "São Tomé e Príncipe" OR Angola OR Benin OR Botswana OR "Burkina Faso" OR Burundi OR "Cape Verde" OR "Cabo Verde" OR Cameroon OR "Central African Republic" OR Chad OR Comoros OR Congo OR "Democratic Republic of Congo" OR "DRC " OR "Cote d'Ivoire" OR "Ivory Coast" OR Djibouti OR Egypt OR "Equatorial Guinea" OR Eritrea OR Eswatini OR Swaziland OR Ethiopia OR Gabon OR Gambia OR Ghana OR Guinea OR "Guinea-Bissau" OR Kenya OR Lesotho OR Liberia OR Libya OR Madagascar OR Malawi OR Mali OR Mauritania OR Mauritius OR Morocco OR Mozambique OR Namibia OR Niger OR Nigeria OR Rwanda OR "Sao Tome and Principe" OR Senegal OR Seychelles OR "Sierra Leone" OR Somalia OR (South* Africa) OR "South Sudan" OR Sudan OR Tanzania OR Togo OR Tunisia OR Uganda OR Zambia OR Zimbabwe)).af and English.lg and "2003 – 2023.yr.

Ovid (Medline):

(("Climate change" OR climate OR "climate risk" OR flood* OR "extreme weather" OR "extreme temperature" OR "extreme rainfall" OR drought OR cyclone) and (vulnerability OR disaster OR susceptibility) and (Africa OR Algeria OR "São Tomé e Príncipe" OR Angola OR Benin OR Botswana OR "Burkina Faso" OR Burundi OR "Cape Verde" OR "Cabo Verde" OR Cameroon OR "Central African Republic" OR Chad OR Comoros OR Congo OR "Democratic Republic of Congo" OR "DRC " OR "Cote d'Ivoire" OR "Ivory Coast" OR Djibouti OR Egypt OR "Equatorial Guinea" OR Eritrea OR Eswatini OR Swaziland OR Ethiopia OR Gabon OR Gambia OR Ghana OR Guinea OR "Guinea-Bissau" OR Kenya OR Lesotho OR Liberia OR Libya OR Madagascar OR Malawi OR Mali OR Mauritania OR Mauritius OR Morocco OR Mozambique OR Namibia OR Niger OR Nigeria OR Rwanda OR "Sao Tome and Principe" OR Senegal OR Seychelles OR "Sierra Leone" OR Somalia OR (South* Africa) OR "South Sudan" OR Sudan OR Tanzania OR Togo OR Tunisia OR Uganda OR Zambia OR Zimbabwe)).af and English.lg and "2003 – 2023.yr.

Web of Science:

(((TS=("Climate change" OR climate OR "climate risk" OR flood* OR "extreme weather" OR "extreme temperature" OR drought OR cyclone)) AND TS=(vulnerability OR disaster OR susceptibility)) AND TS=((Africa OR Algeria OR "São Tomé e Príncipe" OR Angola OR Benin OR Botswana OR "Burkina Faso" OR Burundi OR "Cape Verde" OR "Cabo Verde" OR Cameroon OR "Central African Republic" OR Chad OR Comoros OR Congo OR "Democratic Republic of Congo" OR "DRC " OR "Cote d'Ivoire" OR "Ivory Coast" OR Djibouti OR Egypt OR "Equatorial Guinea" OR Eritrea OR Eswatini OR Swaziland OR Ethiopia OR Gabon OR Gambia OR Ghana OR Guinea OR "Guinea-

Bissau" OR Kenya OR Lesotho OR Liberia OR Libya OR Madagascar OR Malawi OR Mali OR Mauritania OR Mauritius OR Morocco OR Mozambique OR Namibia OR Niger OR Nigeria OR Rwanda OR "Sao Tome and Principe" OR Senegal OR Seychelles OR "Sierra Leone" OR Somalia OR (South* Africa) OR "South Sudan" OR Sudan OR Tanzania OR Togo OR Tunisia OR Uganda OR Zambia OR Zimbabwe))) AND PY=(2003-2023)

Limiter – Language: English

EBSCOhost (CINAHL):

(“Climate change” OR climate OR “climate risk” OR flood* OR “extreme weather” OR “extreme temperature” OR “extreme rainfall” OR drought OR cyclone) AND (vulnerability OR disaster OR susceptibility) AND (Africa OR Algeria OR "São Tomé e Príncipe" OR Angola OR Benin OR Botswana OR "Burkina Faso" OR Burundi OR "Cape Verde" OR "Cabo Verde" OR Cameroon OR "Central African Republic" OR Chad OR Comoros OR Congo OR "Democratic Republic of Congo" OR "DRC " OR "Cote d'Ivoire" OR "Ivory Coast" OR Djibouti OR Egypt OR "Equatorial Guinea" OR Eritrea OR Eswatini OR Swaziland OR Ethiopia OR Gabon OR Gambia OR Ghana OR Guinea OR "Guinea-Bissau" OR Kenya OR Lesotho OR Liberia OR Libya OR Madagascar OR Malawi OR Mali OR Mauritania OR Mauritius OR Morocco OR Mozambique OR Namibia OR Niger OR Nigeria OR Rwanda OR "Sao Tome and Principe" OR Senegal OR Seychelles OR "Sierra Leone" OR Somalia OR (South* Africa) OR "South Sudan" OR Sudan OR Tanzania OR Togo OR Tunisia OR Uganda OR Zambia OR Zimbabwe)

Limiters - Publication Date: 20030101-20231231; Language: English

EBSCOhost (Medline):

(“Climate change” OR climate OR “climate risk” OR flood* OR “extreme weather” OR “extreme temperature” OR “extreme rainfall” OR drought OR cyclone) AND (vulnerability OR disaster OR susceptibility) AND (Africa OR Algeria OR "São Tomé e Príncipe" OR Angola OR Benin OR Botswana OR "Burkina Faso" OR Burundi OR "Cape Verde" OR "Cabo Verde" OR Cameroon OR "Central African Republic" OR Chad OR Comoros OR Congo OR "Democratic Republic of Congo" OR "DRC " OR "Cote d'Ivoire" OR "Ivory Coast" OR Djibouti OR Egypt OR "Equatorial Guinea" OR Eritrea OR Eswatini OR Swaziland OR Ethiopia OR Gabon OR Gambia OR Ghana OR Guinea OR "Guinea-Bissau" OR Kenya OR Lesotho OR Liberia OR Libya OR Madagascar OR Malawi OR Mali OR Mauritania OR Mauritius OR Morocco OR Mozambique OR Namibia OR Niger OR Nigeria OR Rwanda OR "Sao Tome and Principe" OR Senegal OR Seychelles OR "Sierra Leone" OR Somalia OR (South* Africa) OR "South Sudan" OR Sudan OR Tanzania OR Togo OR Tunisia OR Uganda OR Zambia OR Zimbabwe)

Limiters - Publication Date: 20030101-20231231; Language: English

Appendix III: Regions/Countries covered in various articles

Regions / Countries Summary		
	**Region / Country of Study**	**No. of Studies**
Country-specific studies	Ethiopia	16
	South Africa	14
	Nigeria	8
	Ghana	7
	Kenya	5
	Zimbabwe	5
	Malawi	3
	Botswana	2
	Benin	2
	Cameroon	2
	Mozambique	2
	The Gambia	2
	Togo	2
	Uganda	2
	Algeria	1
	Chad	1
	Cote D'Ivoire	1
	Egypt	1
	Lesotho	1
	Mali	1
	Namibia	1
	Senegal	1
	Somalia	1
	Sudan	1
	Tanzania	1
Regional studies	Africa Region	4
	Sub-Saharan Africa Region	2
	West Africa Region	2
	Central Sahel River Basin Region	1
	Middle East and North Africa (MENA)	1
Global studies	All continents including Africa	1
	**Total**	**94**

Footnote: *The articles were grouped into country-specific studies – focused on a particular country or an area within the country, regional studies – focused on various countries within the region and global studies – focused on countries within and beyond Africa*

Appendix IV: Summary of vulnerability models

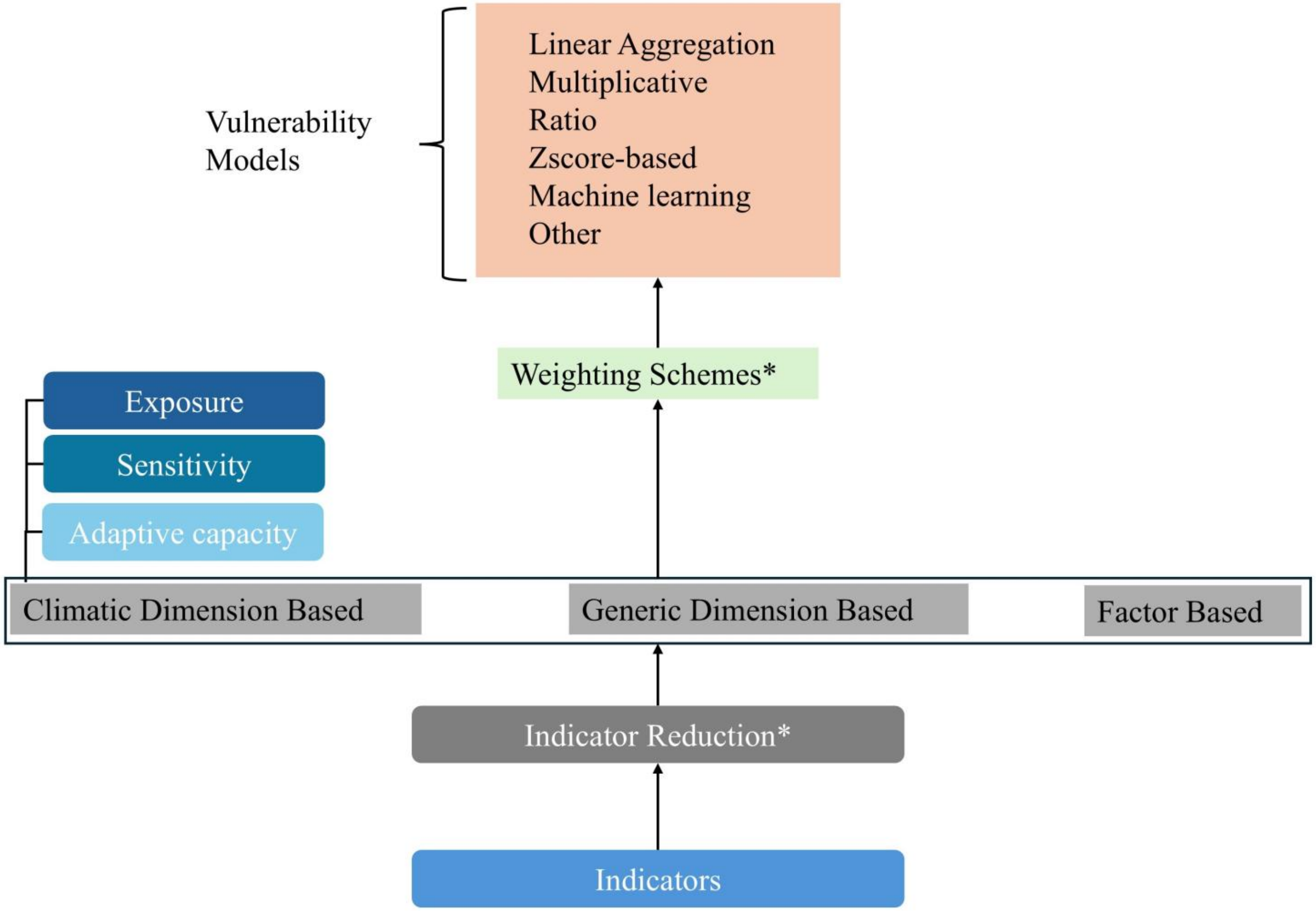

Footnote: *Vulnerability models considered in the review. The * on indicator reduction and weighting schemes means that not all the vulnerability models considered these techniques. "Other" in the methods list highlights an article that used a specific organization-based model, but the equation of the model is not clear. "Generic Dimension Based" group focused on models that used a domain-based approach different from the climate domains. Factor based means the indicators were not grouped in the vulnerability assessment.*

Appendix V: Vulnerability index models highlighted in the review

No.	Model	Grouping	Domain grouping	Domain aggregation method	Vulnerability Function	Citation
1	Linear aggregation	Factor			$\boldsymbol{V = \sum_{i=1}^{n} w_i v_i}$<br>Where: w = equal weighting factor for all variables or indicators; v = each factor or indicator	(1–16)
					$\boldsymbol{V} = \frac{1}{n}\boldsymbol{\sum_{i=1}^{n} w_i * v_i}$<br>Where: w = equal weighting factor for all variables or indicators; v = each factor or indicator; n = sum of indicators	(17–19)
		Domain	Climatic domain (E, S, AC)	Average method:<br>$D = \frac{\sum_{i=1}^{n} \boldsymbol{W_{mi} M_{li}}}{\sum_{i=1}^{n} \boldsymbol{W_{mi}}}$<br>Where: M_{li} are the major subcomponents and W_{mi} are the weights of each major sub-component	$\boldsymbol{V = (E + S) - AC}$	(20,21)
					$\boldsymbol{V = \sum \frac{[(E + S) - AC]}{n}}$<br>Where: n = no. of farmers interviewed	(22)
				Average method:<br>$\boldsymbol{CI = \frac{\sum_{ei=1}^{n} W_j Y_{ij}}{n}}$<br>Where: CI is the composite vulnerability index of exposure factor. Wj is the weight a single indicator, ei is exposure indicators; Yij is the normalised value of exposure indicator; n is the number of indicators	$\boldsymbol{V = (E + S) - AC}$	(23)
				Average method/ arithmetic mean:<br>$\boldsymbol{f} = \frac{1}{n}\sum I_{Sc}$<br>Where: f is either exposure (E), sensitivity (S) or adaptive (A) factor, and n is the total number of indicators for the factor	$\boldsymbol{V = (E + S) - AC}$	(24)
					$\boldsymbol{V = \frac{S + AC}{n}}$<br>Where: n = no. of components or compound indices	(25,26)
					$\boldsymbol{V = \frac{1}{3}(E + S + LoAC)}$	(27,28)
					**Average to produce V/ arithmetic mean of E, S, AC**	(29,30)

					$V = \sum_{i=1}^{5} \frac{1}{2}(\left(\frac{1}{2}\left(\frac{Harvested\ area_i}{Harvested\ area\ total} xS + E\right)\right) + AC)$	(31)
				Average method- combination of crop yield indices at both national and subnational scale (specific equations for E, S and AC based on indicators used in crop yield)	$V = (E + S) - AC$	(32,33)
				Average method $E = \frac{\sum_{i=1}^{n} N_{sub,i} E_{sub,i}}{\sum_{i=1}^{n} N_{sub,i}}$ Where: E= Exposure to drought; $N_{sub,i}$ = number of sub-indices within the main index, and $E_{sub,i}$ = the average score of main index for exposure to droughts.	$V = (E + S) - AC$	(34)
				Linear aggregation/additive $V^{SVI} = \sum_{i=1}^{n} W_i^{svi} v_i^{svi}$	$V = AC - (E + S)$	(35,36)
					$V = E + S + (1 - AC)$	(37)
					$V = E + S + (\frac{1}{LoAC})$	(38)
					Additive: $V = f(E, S, AC)$	(39)
					**$V = f(SU, AC)$ – weighted linear combination**	(40)
					V= $\frac{1}{3}$ $[E + S + (1 - AC)]$	(41–43)
					$V = \frac{(PVI + SVI)}{2}$ Where: PVI = physical vulnerability index; SVI = social vulnerability index	(44)
					**Weighted sum of E, S, AC**	(45)
					**Mean aggregate of E, S, AC**	(46)
					$V = \frac{\frac{E + S}{2} + (1 - AC)}{2}$	(47)
				Zscore method	$V = AC - (E + S)$	(48,49)
					$V = (E + S) - AC$	(50)
				Other: Combined into integrated index	$V = (E + S) - AC$	(51,52)

			Generic domains	PCA	$\boldsymbol{V = \sum [PC_1, PC_2, PC_3 \ldots PC_n]}$<br>Where: PC = principal components	(53–57)
				Average method:<br>$\boldsymbol{f = \sum_{i=1}^{n} w_i v_i}$	$\boldsymbol{V = \sum_{k=1}^{n} W_k\, C_{i,k}}$<br>Where: W_k = weights assigned for the k component; $C_{i,k}$ = components	(58,59)
				Average method/ arithmetic mean:<br>$f = \frac{1}{n} \sum I_{Sc}$	$\boldsymbol{V = f.[\frac{1}{n}(M_{DF} + M_{EF} + M_{SF})]}$<br>Where: M_{DF} = index values of the demographic factors; M_{EF} = index values of the economic factors; M_{SF} = index values of the social factors; n = number of factors (major components) of social vulnerability	(60)
					$\boldsymbol{Vul_i^o = (Vul_i^{Phy} + Vul_i^{Soc} + Vul_i^{Eco} + Vul_i^{Env})/4}$<br>Where: are respectively the average values of each $(Vul_i^{Phy} + Vul_i^{Soc} + Vul_i^{Eco} + Vul_i^{Env})$source of physical vulnerability index , social vulnerability index ,<br>economic vulnerability index and, environmental vulnerability index for the municipality	(61)
					$\boldsymbol{V = \sum_{k=1}^{n} W_k\, C_{i,k}}$<br>Where: W_k = weights assigned for the k component; $C_{i,k}$ = components	(62)
					$\boldsymbol{V = \frac{\sum_{i=1}^{n} w_i B_i}{\sum_{i=1}^{n} W_i}}$<br>Where: $B_1 \ldots B_n$ = indicating baskets; $w_1 \ldots w_n$ = number of indicators in each basket	(63)
					$\boldsymbol{V = \frac{1}{4}}$**(C+P+H+G)**<br>Where: C = climate-related hazard exposure; P = population density; H = household and community resilience; G = governance	(64)
					$\boldsymbol{V = f[F, W][C, S, R][E]}$<br>Where: F = floor elevation W = material used for wall construction and its present nature C = school compound flatness S = soil type R = type of road to the school and distance from asphalt road E = evident effort by school to control or mitigate floods on school property. (Additive function)	(65)
					**Average approach**	(66)

2	Multiplicative model	Domain	Climatic domain (E, S, AC)	Average method: $D = \frac{\sum_{i=1}^{n} \boldsymbol{W_{mi} M_{li}}}{\sum_{i=1}^{n} \boldsymbol{W_{mi}}}$ Where: M_{li} are the major subcomponents and W_{mi} are the weights of each major sub-component	$\boldsymbol{V = (E - AC)xS}$	(67–80)
				Linear aggregation/additive: $V^{SVI} = \sum_{i=1}^{n} W_i^{svi} v_i^{svi}$	$\boldsymbol{V = E\,(\frac{1}{2}\,(S + LoAC))}$	(81)
3	Ratio model	Factor			$V = \sqrt[2]{\frac{G + SCR + BS + E + SLA + H_s + TR}{7}}$ Where: G = geomorphology; SCR = shoreline change rate; BS = beach slope; E = elevation; SLA = sea level anomaly; H_s = significant mean wave height; TR = tidal range	(82)
		Domain	Climatic domain (E, S, AC)	Linear aggregation/additive	$\boldsymbol{V = \frac{E\,x\,S}{Ac}}$	(83–85)
				Average method: $f = \frac{1}{n}\sum I_{Sc}$ Where: f is either exposure (E), sensitivity (S) or adaptive (A) factor, and n is the total number of indicators for the factor	$\boldsymbol{V = \frac{E\,x\,S}{Ac}}$	(86,87)
				Average method: Specific functions provided for landscape E, S and AC	$\boldsymbol{V = \sqrt{\frac{E\,x\,S}{1 + AC}}}$	(88)
				Other: Specific aggregation method of the components not provided	$\mathbf{V} = \frac{[(\mathbf{E} + \mathbf{S})]}{\boldsymbol{AC}}$	(89)
			Generic domains	Average method	$\boldsymbol{V = \frac{\sum_{p=1}^{n} S_{x,p,c}}{\sum_{p=1}^{n} T_{x,p,c}}}$ Where: S = score (ND-GAIN or WRI) associated with the country of production p; T = amount (MT) of traded crop x; c = consuming country (The Gambia); n = number of supplying countries	(90)

4	Zscore based model	Factor			$$CDVI_t = \frac{Y_t - Mean\ (Y)}{STD\ (Y)}$$ Where: CDVI = crop drought vulnerability index; Y_t = yield residuals for year t; Mean(Y) and STD(Y) are the mean and standard deviation of the yield residuals (Y)	(91)
					$$V = \sum_{i=1}^{k} [b_i\ (a_{ji} - x_i)]/S_i$$ Where: v is the vulnerability index, b is the weights from PCA 1, a is the indicator value, x is the mean indicator value, s is the standard deviation of the indicators, i is the indicators and j is the specific province	(92)
4	Machine learning	Factor			Random forest, K-nearest neighbours, support vector machine and naÃ¯ve Bayes	(93)
5	Other	Domain	Generic domains		Drought vulnerability evaluated using household vulnerability index developed by Food Agriculture and Natural Resources Policy Analysis Network (FANRPAN) (equation not provided)	(94)

Footnote: *Summary of vulnerability index equations. Each type of model was grouped based on factor, climatic domain and other domains. Factor based considered the individual indicators, climatic domains considered the exposure, sensitivity and adaptive capacity as the major components while generic domains considered varied groupings separate from climatic domains as the major components. Methods used to obtain the different domains is also provided in the domain aggregation methods column. Abbreviations: Exposure (E), Sensitivity (S), Adaptive capacity (AC), Vulnerability index (V)*

Appendix VI: Vulnerability index and the various major sub-component of the indicators

Vulnerability index	Dimensions /Major sub-components
Children climate	Adaptive capacity (Education, Poverty, WASH, Communication assets and social protection); Sensitivity (Child health and nutrition)
Climate	Adaptive capacity (Institutional development, Natural capital, Social capital, Education, Financial capital, Human capital, Physical capital, Income level diversification); Exposure (Climate, Climate hazards, Climate variability); Sensitivity (Livestock farming, Culture, Consumption, Natural resources, Agricultural, Poverty and Hunger, Health, Demographic, Disaster related, Food security
Climate risk	Adaptive capacity, Exposure, Sensitivity
Climate security	Governance and political violence, Population, Household and community resilience, Physical exposure
Climate-water conflict	Adaptive capacity (Socio-demographic, Physical/natural assets, Livelihood income strategies, Social/political network); Exposure (Climate variability, Water conflict); Sensitivity (Lake water variability
Coastal community	Adaptive capacity; Exposure; Sensitivity
Community	Adaptive capacity; Exposure; Sensitivity
Crop drought	Infrastructure, Resources, Human, Governance, Economic
Crop yield	Adaptive capacity (Socio-economic); Exposure (Climate drivers of drought); Sensitivity (Crop yields)
Drought	Adaptive capacity; Exposure; Sensitivity
	Human and civic resource, Economic, Renewable natural capital, Infrastructure and Technology
Ecological	Adaptive capacity; Exposure; Sensitivity
Farmers	Adaptive capacity; Exposure; Sensitivity
Farmers household	Adaptive capacity; Exposure; Sensitivity
Farming	Adaptive capacity (Social capital, Physical capital, Human capital, Financial capital); Exposure; Sensitivity
Flood	Adaptive capacity (Social, Economic, Physical, Environmental); Exposure (Physical/environmental, Social, Structure population, Economic, Physical); Sensitivity (Physical/environmental, Social, Structure population, Economic, Physical)
	Social, Morphometric parameters, Technological, Flood causative, Ecological, Exposure to hazards, Method of mitigation, Sanitation and disease, Income, Economic
Gender	Adaptive capacity; Exposure; Sensitivity (Socio-economic, Agro-environmental, Water)
Health	Adaptive capacity (Physical capital, Financial capital, Social capital, Human capital); Exposure (Climate); Sensitivity (Natural capital)
Household	Adaptive capacity; Exposure; Sensitivity
	Natural assets, Human capital, Social assets, Physical assets, Financial assets
Integrated coastal	Physical, Socio-economic
Landscape	Adaptive capacity; Exposure; Sensitivity
Livelihood	Adaptive capacity (Natural capital, Assets, Training and support, Livelihood diversification, Literacy, Perception and willingness, Financial capital, Socio-demographic, Social capital, Human capital, Physical capital, Farm inputs, Demographic, Social, Sustainable livelihood capital/assets, Social institutions or networks, Livelihood strategy, Water, Access to services, Wealth, Infrastructure, Social networks, Food); Exposure (Natural disaster, Climate hazards, Climate variability, Drought, Water variability, Climate, Weather and climate events, Water variability, Natural hazards); Sensitivity (Natural capital, Runoff, Health security, Socio-economic, Production, Biophysical environment, Health, Food security, Stability, Food and nutrition, Water security, Surface water storage, Agricultural, Biophysical, Income and food access, Water storage capacity)
Maize yield	Adaptive capacity; Exposure; Sensitivity
Multi-hazard	Adaptive capacity (Physical, Management and institutional, Social, Economic); Exposure (Economic, Physical); Sensitivity (Economic, Physical, Social)
Multivariate agricultural drought	Adaptive capacity (Socio-economic); Exposure (Climate); Sensitivity (Physiographic and environmental, Biophysical drought)
Population	Adaptive capacity; Exposure; Sensitivity
Rural community	Adaptive capacity (Physical planning and engineering, Economic capacity, Management and institutional capacity, Societal capacity); Exposure (Structure, Population economy); Sensitivity (Social, Physical/demographic factor, Environmental, Economic)
Rural household	Adaptive capacity; Exposure; Sensitivity
Sectoral vulnerability	Human habitat, Ecosystem services, Infrastructure, Water, Health, Food
Social	Adaptive capacity; Exposure; Sensitivity

	Population density, Vulnerability preparedness, Access to resources, Land-related, Access and distance to infrastructure and services, Demographic, Household characteristics, Employment, Economic, Income-related, Access to services, Land use and green space, Rurality, Socio-economic, Social, Climate change related, Family structure, Vulnerable populations
Socio-ecological	Ecological, Economic, Infrastructure, Social
West Sudanian Community	Adaptive capacity (Ecosystem robustness, Coping capacity); Exposure (Ecological, Social system); Sensitivity (Poverty and dependency, Housing conditions, Health and Nutrition, Public infrastructure)

Appendix VII: Climatic hazards and extreme weather events

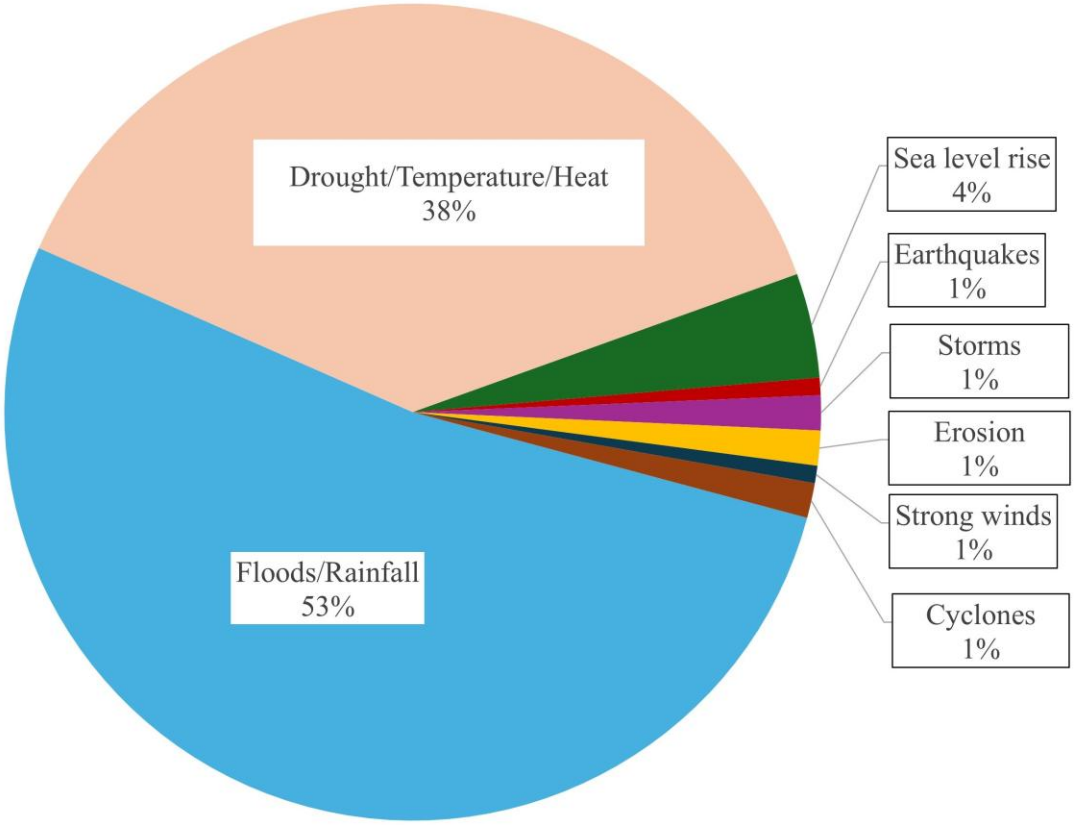

Footnote: Climatic hazards and extreme weather events discussed in the various articles.

References

1. Jordaan A, Bahta YT, Phatudi-Mphahlele B. Ecological vulnerability indicators to drought: Case of communal farmers in Eastern Cape, South Africa. Jamba (Potchefstroom, South Africa). 2019;11(1):591.

2. Bahta Y, Lombard W. Nexus between Social Vulnerability and Resilience to Agricultural Drought amongst South African Smallholder Livestock Households. Atmosphere. 2023;14(5).

3. Bahta Y. Social vulnerability to agricultural drought: Insights from Northern Cape, South Africa. Scientific African. 2022;17.

4. Muyambo F, Jordaan AJ, Bahta YT. Assessing social vulnerability to drought in South Africa: Policy implication for drought risk reduction. Jamba (Potchefstroom, South Africa). 2017;9(1):326.

5. Badmos B, Adenle A, Agodzo S, Villamor G, Asare-Kyei D, Amadou L, et al. Micro-level social vulnerability assessment towards climate change adaptation in semi-arid Ghana, West Africa. Environment Development and Sustainability. 2018;20(5):2261–79.

6. Dintwa KF, Letamo G, Navaneetham K. Measuring social vulnerability to natural hazards at the district level in Botswana. Jamba (Potchefstroom, South Africa). 2019;11(1):447.

7. Abdrabo KI, Kantoush SA, Saber M, Sumi T, Habiba OM, Elleithy D, et al. Integrated Methodology for Urban Flood Risk Mapping at the Microscale in Ungauged Regions: A Case Study of Hurghada, Egypt. Remote Sensing. 2020;12(21).

8. Mokhtari E, Mezali F, Abdelkebir B, Engel B. Flood risk assessment using analytical hierarchy process: a case study from the Cheliff-Ghrib watershed, Algeria. Journal of Water and Climate Change. 2023;14(3):694–711.

9. Eguaroje O, Alaga T, Ogbole J, Omolere S, Alwadood J, Kolawole I, et al. Flood vulnerability assessment of Ibadan city, Oyo state, Nigeria. World Environment. 2015;5(4):149–59.

10. Mohamed A, Worku H. Urban land cover and morphometric analysis for flash flood vulnerability mapping and riparian landscape conservation in Kebena River watershed, Addis Ababa. Appl Geomat. 2021;13(1):15–28.

11. Osman S, Das J. GIS-based flood risk assessment using multi-criteria decision analysis of Shebelle River Basin in southern Somalia. SN Applied Sciences. 2023;5(5).

12. Alimi S, Andongma T, Ogungbade O, Senbore S, Alepa V, Akinlabi O, et al. Flood vulnerable zones mapping using geospatial techniques: Case study of Osogbo Metropolis, Nigeria. Egyptian Journal of Remote Sensing and Space Sciences. 2022;25(3):841–50.

13. Durowoju O, Ologunorisa T, Akinbobola A. Assessing agricultural and hydrological drought vulnerability in a savanna ecological zone of Sub-Saharan Africa. Natural Hazards. 2022;111(3):2431–58.

14. Desalegn H, Mulu A. Flood vulnerability assessment using GIS at Fetam watershed, upper Abbay basin, Ethiopia. Heliyon. 2021;7(1):e05865.

15. Leta BM, Adugna D. Characterizing the level of urban Flood vulnerability using the social-ecological-technological systems framework, the case of Adama city, Ethiopia. Heliyon. 2023;9(10):e20723.

16. Musungu K, Motala S, Smit J. Using multi-criteria evaluation and GIS for flood risk analysis in informal settlements of Cape Town: The case of graveyard pond. South African Journal of Geomatics. 2012;1(1):92–108.

17. Lottering S, Mafongoya P, Lottering R. Assessing the social vulnerability of small-scale farmer's to drought in uMsinga, KwaZulu-Natal. International Journal of Disaster Risk Reduction. 2021;65.

18. Ouma YO, Tateishi R. Urban Flood Vulnerability and Risk Mapping Using Integrated Multi-Parametric AHP and GIS: Methodological Overview and Case Study Assessment. Water. 2014;6(6):1515–45.

19. Ogarekpe N, Obio E, Tenebe I, Emenike P, Nnaji C. Flood vulnerability assessment of the upper Cross River basin using morphometric analysis. Geomatics Natural Hazards & Risk. 2020;11(1):1378–403.

20. Dzoga M, Simatele D, Munga C. Assessment of ecological vulnerability to climate variability on coastal fishing communities: A study of Ungwana Bay and Lower Tana Estuary, Kenya. Ocean & coastal management. 2018;163:437–44.

21. Mekonen A, Berlie A. Rural households' livelihood vulnerability to climate variability and extremes: a livelihood zone-based approach in the Northeastern Highlands of Ethiopia. Ecological Processes. 2021;10(1).

22. Acheampong E, Ozor N, Owusu E. Vulnerability assessment of Northern Ghana to climate variability. Climatic Change. 2014;126(1–2):31–44.

23. Kissi AE, Abbey GA, Agboka K, Egbendewe A. Quantitative assessment of vulnerability to flood hazards in downstream area of Mono Basin, South-Eastern Togo: Yoto District. Journal of Geographic Information System. 2015;7(6):607–19.

24. Yankson P, Owusu A, Owusu G, Boakye-Danquah J, Tetteh J. Assessment of coastal communities' vulnerability to floods using indicator-based approach: a case study of Greater Accra Metropolitan Area, Ghana. Natural Hazards. 2017;89(2):661–89.

25. Samuels M, Masubelele M, Cupido C, Swarts M, Foster J, De Wet G, et al. Climate vulnerability and risks to an indigenous community in the arid zone of South Africa. Journal of Arid Environments. 2022;199.

26. Rees N. The Climate Crisis Is a Child Rights Crisis: Introducing the Children's Climate Risk Index. UNICEF. 2021;

27. Oyedele P, Kola E, Olorunfemi F, Walz Y. Understanding Flood Vulnerability in Local Communities of Kogi State, Nigeria, Using an Index-Based Approach. Water. 2022;14(17).

28. U.S. Agency for International Development (USAID). Mali Climate Vulnerability Mapping [Internet]. [cited 2025 Jan 29]. Available from: https://www.usaid.gov/sites/default/files/2022-05/MALI%20CLIMATE%20VULNERABILITY%20MAPPING.pdf

29. de Sherbinin A, Chai-Onn T, Jaiteh M, Mara V, Pistolesi L, Schnarr E, et al. Data Integration for Climate Vulnerability Mapping in West Africa. ISPRS International Journal of Geo-Information. 2015;4(4):2561–82.

30. Namdar R, Karami E, Keshavarz M. Climate Change and Vulnerability: The Case of MENA Countries. ISPRS International Journal of Geo-Information. 2021;10(11).

31. Parker L, Bourgoin C, Martinez-Valle A, Laderach P. Vulnerability of the agricultural sector to climate change: The development of a pan-tropical Climate Risk Vulnerability Assessment to inform sub-national decision making. PloS one. 2019;14(3):e0213641.

32. Epule T. Recent Patterns of Exposure, Sensitivity, and Adaptive Capacity of Selected Crops in Cameroon. Agriculture-Basel. 2021;11(6).

33. Epule TE, Chehbouni A, Dhiba D, Molua EL. A regional stocktake of maize yield vulnerability to droughts in the Horn of Africa. Environmental monitoring and assessment. 2023;196(1):76.

34. Tessema K, Haile A, Nakawuka P. Vulnerability of community to climate stress: An indicator-based investigation of Upper Gana watershed in Omo Gibe basin in Ethiopia. International Journal of Disaster Risk Reduction. 2021;63.

35. Attipo R, Emvoulou I, Coffi A. Climate change and urban stormwater: vulnerability analysis of the 2010 floods in Lomé, Togo. Frontiers in Climate. 2023;5.

36. Akukwe TI, Ogbodo C. Spatial Analysis of Vulnerability to Flooding in Port Harcourt Metropolis, Nigeria. 2015;

37. Asare-Kyei D, Renaud FG, Kloos J, Walz Y, Rhyner J. Development and validation of risk profiles of West African rural communities facing multiple natural hazards. PloS one. 2017;12(3):e0171921.

38. Erena SH, Worku H. Urban flood vulnerability assessments: the case of Dire Dawa city, Ethiopia. Nat Hazards. 2019;97(2):495–516.

39. Macharia D, Kaijage E, Kindberg L, Koech G, Ndungu L, Wahome A, et al. Mapping Climate Vulnerability of River Basin Communities in Tanzania to Inform Resilience Interventions. Sustainability. 2020;12(10).

40. Kienberger S. Spatial modelling of social and economic vulnerability to floods at the district level in Búzi, Mozambique. Natural Hazards. 2012;64:2001–19.

41. Adeloye A, Mwale F, Dulanya Z. A metric-based assessment of flood risk and vulnerability of rural communities in the Lower Shire Valley, Malawi. In 2015. p. 139–45.

42. Mwale FD, Adeloye AJ, Beevers L. Quantifying vulnerability of rural communities to flooding in SSA: A contemporary disaster management perspective applied to the Lower Shire Valley, Malawi. International journal of disaster risk reduction. 2015;12:172–87.

43. Kamanga T, Tantanee S, Mwale F, Buranajarukorn P. A Multi Hazard Perspective in Flood and Drought Vulnerability: Case Study of Malawi. Geographia technica. 2020;15(1):132–42.

44. Charuka B, Angnuureng D, Brempong E, Agblorti S, Agyakwa K. Assessment of the integrated coastal vulnerability index of Ghana toward future coastal infrastructure investment plans. Ocean & Coastal Management. 2023;244.

45. Membele G, Naidu M, Mutanga O. Application of analytic network process (ANP), local and indigenous knowledge in mapping flood vulnerability in an informal settlement. Natural Hazards. 2023;

46. Balogun V, Onokerhoraye A. Climate change vulnerability mapping across ecological zones in Delta State, Niger Delta Region of Nigeria. Climate Services. 2022;27.

47. Wassie S, Mengistu D, Birlie A, Waktola D. Drought-induced agricultural livelihood vulnerability: Livelihood-based comparative analysis in Northeast highlands of Ethiopia. Cogent Food & Agriculture. 2023;9(1).

48. Fenta M, Jordaan A, Melka Y. Vulnerability of Southern Afar pastoralists to climate variability and change, Ethiopia (vol 11, a575, 2019). Jamba-Journal of Disaster Risk Studies. 2019;11.

49. Mekuyie M. Vulnerability of rural households to climate-induced shocks in Lokka Abaya district, Sidama zone, southern Ethiopia. Jamba (Potchefstroom, South Africa). 2021;13(1):1051.

50. Mugagga F, Nakanjakko N, Nakileza B, Nseka D. Vulnerability of smallholder sorghum farmers to climate variability in a heterogeneous landscape of south-western Uganda. Jamba (Potchefstroom, South Africa). 2020;12(1):849.

51. Jiri O, Mafongoya P, Chivenge P. Contextual vulnerability of rainfed crop-based farming communities in semi-arid Zimbabwe A case of Chiredzi District. International Journal of Climate Change Strategies and Management. 2017;9(6):777–89.

52. Munyai R, Chikoore H, Musyoki A, Chakwizira J, Muofhe T, Xulu N, et al. Vulnerability and Adaptation to Flood Hazards in Rural Settlements of Limpopo Province, South Africa. Water. 2021;13(24).

53. Dintwa K, Letamo G, Navaneetham K. Quantifying social vulnerability to natural hazards in Botswana: An application of cutter model. International Journal of Disaster Risk Reduction. 2019;37.

54. Mavhura E, Manyena B, Collins AE. An approach for measuring social vulnerability in context: The case of flood hazards in Muzarabani district, Zimbabwe. Geoforum. 2017 Nov 1;86:103–17.

55. Jagarnath M, Thambiran T, Gebreslasie M. Heat stress risk and vulnerability under climate change in Durban metropolitan, South Africa-identifying urban planning priorities for adaptation. Climatic Change. 2020;163(2):807–29.

56. Mavhura E, Manyangadze T. A comprehensive spatial analysis of social vulnerability to natural hazards in Zimbabwe: Driving factors and policy implications. International Journal of Disaster Risk Reduction. 2021;56.

57. Letsie M, Grab S. Assessment of Social Vulnerability to Natural Hazards in the Mountain Kingdom of Lesotho. Mountain Research and Development. 2015;35(2):115–25.

58. Kotzee I, Reyers B. Piloting a social-ecological index for measuring flood resilience: A composite index approach. Ecological Indicators. 2016;60:45–53.

59. Kapuka A, Hlásny T. Social Vulnerability to Natural Hazards in Namibia: A District-Based Analysis. Sustainability. 2020;12(12).

60. Dumenu W.K., Obeng E.A. Climate change and rural communities in Ghana: Social vulnerability, impacts, adaptations and policy implications. Environmental Science and Policy. 2016;55((Dumenu, Obeng) Forestry Research Institute of Ghana, P. O. Box UP 63, Kumasi, Ghana):208–17.

61. Behanzin ID, Thiel M, Szarzynski J, Boko M. GIS-based mapping of flood vulnerability and risk in the Bénin Niger River Valley. International journal of Geomatics and Geosciences. 2016;6(3):1653–69.

62. Naumann G, Barbosa P, Garrote L, Iglesias A, Vogt J. Exploring drought vulnerability in Africa: an indicator based analysis to be used in early warning systems. Hydrology and Earth System Sciences. 2014;18(5):1591–604.

63. Okpara UT, Stringer LC, Dougill AJ. Using a novel climate-water conflict vulnerability index to capture double exposures in Lake Chad. Regional environmental change. 2017;17(2):351–66.

64. Busby J, Smith T, Krishnan N. Climate security vulnerability in Africa mapping 3.0. Political Geography. 2014;43:51–67.

65. Ochola SO, Eitel B, Olago DO. Vulnerability of schools to floods in Nyando River catchment, Kenya. Disasters. 2010;34(3):732–54.

66. Birkmann J, Feldmeyer D, McMillan J, Solecki W, Totin E, Roberts D, et al. Regional clusters of vulnerability show the need for transboundary cooperation. Environmental Research Letters. 2021;16(9).

67. Simane B, Deressa W, Kumie A, Woyessa A, Kaba M, Taye G, et al. Health Vulnerability and Adaptation Strategies to Climate Change in Ethiopia. In 2017. p. 169–83.

68. Hahn MB, Riederer AM, Foster SO. The Livelihood Vulnerability Index: A pragmatic approach to assessing risks from climate variability and change—A case study in Mozambique. Global environmental change. 2009;19(1):74–88.

69. Dechassa C, Simane B, Alamirew B. Farmers' Livelihoods Vulnerability to Climate Variability and Change in Didesa Basin Southern Part of Abay Basin, Ethiopia. In 2017. p. 267–84.

70. Asmamaw M, Mereta S, Beyene E, Ambelu A. Multidimensional livelihood vulnerability analysis in Dinki watershed, central highlands of Ethiopia. Climate and Development. 2020;12(9):814–26.

71. Williams P, Crespo O, Abu M. Assessing vulnerability of horticultural smallholders' to climate variability in Ghana: applying the livelihood vulnerability approach. Environment Development and Sustainability. 2020;22(3):2321–42.

72. Basiru A, Oladoye A, Adekoya O, Akomolede L, Oeba V, Awodutire O, et al. Livelihood Vulnerability Index: Gender Dimension to Climate Change and Variability in REDD. Land. 2022;11(8).

73. Mohmmed A, Li J, Elaru J, Elbashier M, Keesstra S, Artemi C, et al. Assessing drought vulnerability and adaptation among farmers in Gadaref region, Eastern Sudan. Land Use Policy. 2018;70:402–13.

74. Mugandani R, Muziri T, Murewi C, Mugadza A, Chitata T, Sungirai M, et al. Mapping and Managing Livelihoods Vulnerability to Drought: A Case Study of Chivi District in Zimbabwe. Climate. 2022;10(12).

75. Bedeke S, Vanhove W, Wordofa M, Natarajan K, Van Damme P. Vulnerability to climate change among maize-dependent smallholders in three districts of Ethiopia. Environment Development and Sustainability. 2020;22(2):693–718.

76. Opiyo S, Letema S, Opinde G. Characterizing rural households' livelihood vulnerability to climate change and extremes in Migori River Watershed, Kenya. Climate and Development. 2023;

77. Simane B, Zaitchik BF, Foltz JD. Agroecosystem specific climate vulnerability analysis: application of the livelihood vulnerability index to a tropical highland region. Mitigation and adaptation strategies for global change. 2016;21(1):39–65.

78. Asfaw A, Bantider A, Simane B, Hassen A. Smallholder farmers' livelihood vulnerability to climate change-induced hazards: agroecology-based comparative analysis in Northcentral Ethiopia (Woleka Sub-basin). Heliyon. 2021;7(4):e06761.

79. Ntali Y, Lyimo J. Community livelihood vulnerability to drought in semi-arid areas of northern Cameroon. Discover Sustainability. 2022;3(1).

80. Mwadzingeni L, Mugandani R, Mafongoya P. Assessing Vulnerability to Climate Change in Smallholder Irrigation Schemes of Zimbabwe. Sustainability. 2021;13(18).

81. Kablan M, Dongo K, Coulibaly M. Assessment of Social Vulnerability to Flood in Urban Cote d'Ivoire Using the MOVE Framework. Water. 2017;9(4).

82. Mendoza E, Salameh E, Sakho I, Turki I, Almar R, Ojeda E, et al. Coastal flood vulnerability assessment, a satellite remote sensing and modeling approach. Remote Sensing Applications-Society and Environment. 2023;29.

83. Belcore E, Pezzoli A, Calvo A. Analysis of gender vulnerability to climate-related hazards in a rural area of Ethiopia. Geographical Journal. 2020;186(2):156–70.

84. Okaka F, Odhiambo B. Health vulnerability to flood-induced risks of households in flood-prone informal settlements in the Coastal City of Mombasa, Kenya. Natural Hazards. 2019;99(2):1007–29.

85. Munyai RB, Musyoki A, Nethengwe NS. An assessment of flood vulnerability and adaptation: A case study of Hamutsha-Muungamunwe village, Makhado municipality. Jamba (Potchefstroom, South Africa). 2019;11(2):692.

86. Babanawo D, Mattah P, Agblorti S, Brempong E, Mattah M, Aheto D. Local Indicator-Based Flood Vulnerability Indices and Predictors of Relocation in the Ketu South Municipal Area of Ghana. Sustainability. 2022;14(9).

87. Hounkpè J, Badou D, Ahouansou D, Totin E, Sintondji L. Assessing observed and projected flood vulnerability under climate change using multi-modeling statistical approaches in the Oueme River Basin, Benin (West Africa). Regional Environmental Change. 2022;22(4).

88. Guo X, Zhang Z, Zhang X, Bi M, Das P. Landscape vulnerability assessment driven by drought and precipitation anomalies in sub-Saharan Africa. Environmental Research Letters. 2023;18(6).

89. Gomez M, Adelegan O, Ntajal J, Trawally D. Vulnerability to coastal erosion in The Gambia: Empirical experience from Gunjur. International Journal of Disaster Risk Reduction. 2020;45.

90. Hadida G, Ali Z, Kastner T, Carr TW, Prentice AM, Green R, et al. Changes in Climate Vulnerability and Projected Water Stress of The Gambia's Food Supply Between 1988 and 2018: Trading With Trade-Offs. Frontiers in public health. 2022;10(101616579):786071.

91. Kamali B, Abbaspour K, Wehrli B, Yang H. A Quantitative Analysis of Socio-Economic Determinants Influencing Crop Drought Vulnerability in Sub-Saharan Africa. Sustainability. 2019;11(21).

92. Gbetibouo G, Ringler C, Hassan R. Vulnerability of the South African farming sector to climate change and variability: An indicator approach. Natural Resources Forum. 2010;34(3):175–87.

93. Houmma I, El Mansouri L, Gadal S, Faouzi E, Toure A, Garba M, et al. Drought vulnerability of central Sahel agrosystems: a modelling-approach based on magnitudes of changes and machine learning techniques. International Journal of Remote Sensing. 2023;44(14):4262–300.

94. Nembilwi N, Chikoore H, Kori E, Munyai R, Manyanya T. The Occurrence of Drought in Mopani District Municipality, South Africa: Impacts, Vulnerability and Adaptation. Climate. 2021;9(4).
